# A spatially-heterogeneous impact of fencing on the African swine fever wavefront in the Korean wild boar population

**DOI:** 10.1186/s13567-024-01422-7

**Published:** 2024-12-18

**Authors:** Jun-Sik Lim, Timothée Vergne, Eutteum Kim, Claire Guinat, Simon Dellicour, Mathieu Andraud

**Affiliations:** 1 IHAP, Université de Toulouse, INRAE, ENVT, Toulouse, France; 2 Department of Companion Animal Industry, Daegu University, Gyeongsan-si, Republic of Korea; 3 Spatial Epidemiology Lab (SpELL), Université Libre de Bruxelles, Brussels, Belgium; 4 Department of Microbiology, Immunology and Transplantation, Laboratory for Clinical and Epidemiological Virology, Rega Institute, KU Louvain, University of Leuven, Louvain, Belgium; 5 Epidemiology, Health and Welfare Research Unit, Ploufragan‑Plouzané‑Niort Laboratory, Anses, French Agency for Food, Environmental and Occupational Health & Safety, Ploufragan, France

**Keywords:** African swine fever, wild boar, spatial modelling, intervention strategy, South Korea

## Abstract

In October 2019, South Korea’s first case of African swine fever (ASF) was reported in wild boar in the north of the country. Despite the implementation of a 2300 km-long fencing strategy, the ASF wavefront continued to invade southward. Our study aimed to investigate the ASF wavefront dynamics in different regions of South Korea, as well as to assess the effectiveness of the fencing measures on ASF dispersal and wavefront velocity. From the nationwide wild boar surveillance system, we extracted 2661 cases, starting from 2 October 2019 (first detection) to 15 September 2022. The cases were categorised into four main spatiotemporal clusters. The average wavefront velocity over the four clusters was estimated at 0.52 km/week, with the cluster in the eastern part of the Korean peninsula exhibiting the fastest velocity (0.99 km/week) compared to the other clusters (0.44, 0.31, and 0.15 km/week). We hypothesise that these differences are related to different wild boar densities due to heterogeneous habitat suitability. We also found that fencing significantly impacted ASF dispersal in only two of the four main clusters, with no evidence that fencing slowed down the spread of the wavefront in any of the clusters. We argue that this heterogeneity might result from fencing locations being misaligned with the true (and unobserved) wavefront.

**Supplementary Information:**

The online version contains supplementary material available at 10.1186/s13567-024-01422-7.

## Introduction

African swine fever (ASF) is a World Organisation for Animal Health listed disease [1]. It is caused by the African swine fever virus (ASFV), which can infect wild suids including wild boar (*Sus scrofa*), and domestic pigs (*Sus scrofa domesticus*) with a case-fatality rate close to 100% [2]. Since 2007, ASFV genotype II spread across the entire Eurasian continent [3, 4]. While Vietnam has commercialized a vaccine, no universally recognized effective treatment or vaccine has been commercialized in the rest of the world [3].

In September 2019, South Korea reported its first ASF outbreak in domestic pig farms in the northern part of the country, followed in October by the first report in wild boar. In response to these events, Korean authorities started implementing wild boar surveillance and intervention measures, including active carcass search and removal, trapping and hunting by government-employed hunters, local citizens, and soldiers [5]. To improve the sensitivity of wild boar surveillance, different financial incentives were provided for the reporting of ASF-positive wild boar carcasses [5]. Despite the intensity of these measures, it is acknowledged that many wild boar cases have likely remained undetected.

The authorities also built nationwide fences crossing the Korean peninsula to prevent the ASF wavefront from progressing southward into ASF-naive regions [5]. In November 2019, one month after the first detected case, these fences were already about 250 km long. As more cases of ASF were reported in the south of these fences, the authorities responded by building more fences. Although this effort persisted until the end of 2021, ASFV continued to breach the fences and extend its wavefront to the southern region of the county (Additional file 1A). As of October 2023, 3200 wild boar cases were detected, along with 38 domestic pig outbreaks suspected to be mainly due to spillover events from wild boar population [5, 6], threatening the remaining southern regions of the country. This context prompted a discussion about the effectiveness of fencing on ASF wavefront dynamics in South Korea.

Previous studies have assessed the effectiveness of fences to block ASF spread in wild boar in various epidemiological contexts and have found varying scientific evidence of their efficacy [7–9]. In Belgium, it has been shown that the network of barriers that were installed in response to the emergence of ASF in 2018 had a significant impact on both the virus dispersal and the wavefront velocity [10]. A paper using simulations of a model of ASF transmission in a European setting acknowledged that the assessment of fence effectiveness was difficult because of a high uncertainty on some model parameter values, especially the permeability of fences [9]. However, they suggested that fences could be particularly useful in contexts where carcass removal or intensive hunting is challenging. Finally, in the context of South Korea, Han et al. [8] used a simulation model to analyse ASF data in wild boar from October 2019 to June 2020 and suggested that, during that phase, fencing was only partially effective, delaying the progression and giving more time to improve the biosecurity level for domestic pig farms in South Korea. To better understand the dispersion pattern of ASF in South Korea and the effect fencing had on the long run, we applied the methodology developed by Dellicour et al. [10] to all ASF cases in wild boar reported in South Korea between October 2019 and September 2022. In doing so, we estimated the wild boar-mediated ASF wavefront velocity and assessed the impact of fencing on ASF progression.

## Materials and methods

**Figure 1:**
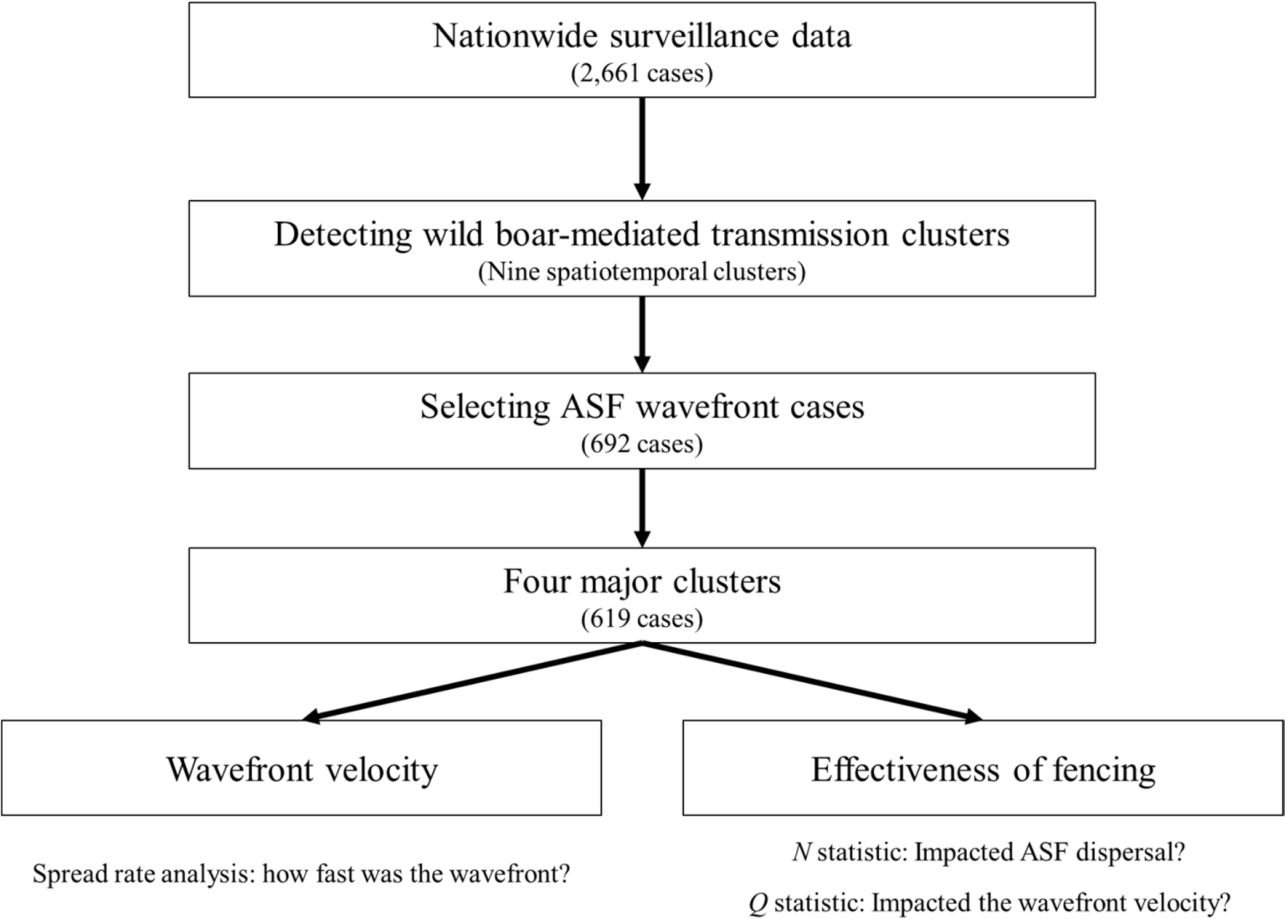
**Workflow to select wild boar-mediated ASF wavefront cases to estimate the wavefront velocity and assess how it was affected by fencing**

To estimate the wavefront velocity and the effectiveness of fencing, several steps were taken, as depicted in Figure 1 and described in the subsequent paragraphs.

### Dataset

We used all wild boar cases reported from nationwide surveillance data operated by the South Korean Ministry of Environment Korea. The study period extended from 2 October 2019, when the first case of ASF was reported in wild boar in South Korea, to 15 September 2022. During that period, a total of 2661 ASF cases were officially reported in wild boar populations. Given that only 25 cases of domestic pig farms have been reported during the study period [6] and that most of them were generally considered to be due to spillover events from wild boars [5], it is likely that ASF outbreaks in domestic pigs played a negligible role in the overall ASF dynamics in wild boar. In this context, we excluded from our analysis the ASF outbreaks reported in domestic pigs and assumed that our approach remained valid for characterising the ASF wavefront in wild boar. We were granted access to the ASF database by the South Korean Ministry of Environment. It included the type of sample (carcass, hunted animal, trapped animal), the date of wild boar detection, the date of diagnosis, and geographical coordinates (longitude and latitude) as well as the estimated date of death for the carcass samples, established by experts from the South Korean Ministry of Environment. This latter assessment was based on the post-mortem examination including carcass quality, and the environmental, ecological, and meteorological aspects surrounding the carcass. Because of the inherent uncertainty in the estimation of the death date, we assessed the sensitivity of our results to a systematic bias in this estimation by also running all the subsequent analyses with a delay between expected death and detection modified by − 50%, − 20%, + 20% and + 50%.

Out of the 2661 ASF cases in wild boar, the majority (90%, 2395 cases) were from carcasses, while 8% (212) and 2% (54) of all cases were from hunted and trapped wild boars, respectively. Regarding the carcasses, the average estimated delay between the date of death and the date of wild boar detection was 13.8 days, with 95% and 50% of the cases being associated with an estimated date of death less than 45 days, and 7 days, respectively. All the analyses presented in this paper were based on the estimated date of death, instead of the date of wild boar detection, because spatially- and temporally- heterogeneous surveillance intensities prevented the detection date from meaning the same thing for different wild boar cases. Using the date of death for whole analysis thus facilitated the understanding of the wavefront dynamics. For the hunted and trapped animals, we considered that the estimated date of death was the date of wild boar detection.

**Figure 2:**
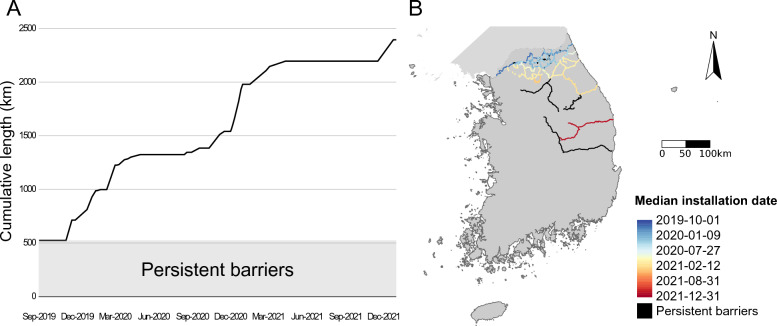
**The temporal patterns of the cumulative length (A) and spatial locations of the installed fences (B)**. Persistent barriers indicate the natural or anthropogenic barriers used for fencing such as cliffs, rivers, highways, etc.

We obtained the geographic location data of all nationwide fences from the South Korean Ministry of Environment and extracted the starting and finishing dates for the installation of each fence from government press releases [11]. Based on these temporal data, we calculated the median installation date between the starting date and finishing date for the installation of each fence. For certain fences, press releases reported the starting installation dates as a time interval. To get conservative results in assessing the effectiveness of fencing, we selected the earliest available date as the starting installation date. This allowed us to reconstruct the dynamics of the continuous construction process of the fences (Figure 2). No information was available on the types of fences that were used.

### Identifying wild boar-mediated transmission clusters

In South Korea, ASF dynamics in wild boar population is influenced by wild boar (or carcass) to wild boar transmission, human activities and potential introductions from North Korea [5]. Given that this study aims to characterize ASF dynamics driven by wild boar, we identified the cluster of cases that were due to wild boar-mediated transmission events. To do so, we first assumed that cases for which the minimum distance from any previous case exceeded 30 km were due to human activities or introductions from North Korea [12]. These resulting “index cases” were then considered as the seed cases for each wild boar-mediated transmission cluster. We then chronologically assigned each subsequent case to the closest existing cluster based on the geographical proximity. To assess the sensitivity of our results to the threshold of 30 km, we also ran the subsequent analyses with thresholds of 20 and 40 km.

### Filtering the wavefront cases

To identify the cases that expanded the frontiers of the areas affected, referred to as the wavefront cases, we used the approach detailed by Dellicour, Desmecht, Paternostre, Malengreaux, Licoppe, Gilbert, Linden and Park [10]. Briefly, for a given cluster, we used all cluster cases estimated to have died, been hunted, or trapped at day *t* to generate a 95% kernel density polygon (KDP) with a resolution of 1 km × 1 km. This KDP was considered as the ASF-affected area at time *t*. All cases of the cluster estimated to have died, been hunted, or trapped at day *t* + *1* and reported outside the previous ASF-affected area were considered to be wavefront cases. We iterated this process for each cluster, starting from the date of death of the cluster index case and continuing until the last case of the cluster was integrated [13].

### Estimating the wavefront velocity

We estimated the wavefront velocity of each cluster by applying a trend surface analysis using the thin plate regression splines interpolation method and a neighbouring spread rate estimator [14]. On a raster of 1 km × 1 km resolution covering the study region, we assigned to each detected cell the estimated date of death of the corresponding wavefront cases. We employed thin spline interpolation to smooth these estimated death dates over the whole study area. On this interpolated surface, we calculated the local slope (week/km) using a 3 × 3 cell window. To avoid the presence of infinite local values, these local slopes were subsequently smoothed by using a 11 × 11 cell smoothing filter [15]. We reversed these smoothed local slopes to obtain wavefront velocity (km/week). The library *fields* in R software was used for this analysis [16]. Finally, we plotted the estimated wavefront velocity for each cluster as a function of the habitat suitability of wild boar at the location of the cases belonging to the corresponding clusters. The habitat suitability values, defined between 0 and 1, were obtained from a previous study [17], which characterised wild boar habitat suitability based on wild boar observation data.

### Assessing the impact of fencing on ASF wavefront

To assess the effectiveness of fencing, we adopted the two analytical frameworks developed in ref. [10] and evaluated the impact of fencing on ASF dispersal and on wavefront velocity. These frameworks were applied to all ASF wavefront cases together, irrespective of their cluster, as well as to each cluster independently.

To assess the impact of fencing on ASF dispersal, we first defined the dispersal vectors as the straight-line segments connecting the seed case to all wavefront cases of its corresponding cluster. We then computed the *N* statistic, defined as the number of times the dispersal vectors crossed a fence that was installed before the estimated date of death of the wavefront cases. As mentioned above, we computed an observed *N* statistic for the entire dataset (referred to as the national-level observed *N* statistic) and one for each cluster (referred to as the cluster-level observed *N* statistics) to assess the impact of fencing on ASF dispersal at the national level and locally in identified clusters, respectively. To test whether the observed *N* statistics were significantly smaller than the ones that would be obtained under a scenario where installed fences had no impact on ASF dispersal we used a permutation approach allowing us to obtain the distribution of the *N* statistics under the null hypothesis [10]. To do so, we ran 200 iterations in which we randomly and independently rotated all dispersal vectors around their corresponding seed case. To ensure that the rotated tip of a dispersal vector remained within the boundaries of South Korea, we re-ran the rotation until the tip was located within South Korea. For each iteration, we re-calculated an *N* statistic, hereafter referred to as a “simulated *N* statistic”, and eventually conducted a one-tail statistical test by estimating a *p*-value equal to the proportion of the simulated *N* statistics that were smaller than the observed *N* statistic. The null hypothesis of this state was thus that the fence did not significantly impact on ASF dispersal from one side of a fence to the other. When the *p*-value was < 0.05, we therefore considered that the observed number of fence-crossing events was significantly smaller than what would have been observed if the fences had no impact on ASF dispersal.

To assess the impact of fencing on the wavefront velocity, we further used the dispersal vectors following the analytical framework proposed by Dellicour et al. [10]. Specifically, we computed the correlation statistic *Q*, calculated as the difference between the coefficients of determinants (${R}^{2}$) from two linear regression models. The first coefficient (${R}_{env}^{2}$) is obtained from the linear regression model *t* ~ *d*_env_, where time duration (*t*) between the seed case and its corresponding case in dispersal vectors is the dependent variable, and environmental distance between those cases (*d*_env_) is the independent variable. *d*_env_ was computed on a geo-referenced grid (raster) with a 1 × 1 km resolution incorporating the location of installed fences. The second coefficient of determination (${R}_{null}^{2}$) is derived from the regression model *t* ~ *d*_null_, where time duration (*t*) is solely regressed against a proxy for geographical distance (*d*_null_). *d*_null_ was computed on the corresponding null raster that is a uniform raster with a value of “1” assigned to all cells. To compute both distances, we employed the path model implemented in the program *Circuitscape* [18] that uses circuit theory to compute the distances as pairwise electric resistances, allowing us to integrate the numerous geographical transmission routes between a seed case and a corresponding case [19]. The statistic *Q* (= ${R}_{env}^{2}-{R}_{null}^{2}$) aims to measure to what extent the fences raster better explains the heterogeneity in velocities than a homogeneous raster that only accounts for geographical distances in the path model. We ensured that both the *Q* statistic and the regression coefficient for *d*_env_ were over zero, so that a positive *Q* statistic would suggest that fencing slows down wavefront velocity. The fences raster was tested as a resistance factor and was obtained by projecting fence features on the null raster by assigning a value of 1 + *k* to each raster cell crossed by a fence. We tested six different values for the scaling parameter *k*, i.e., 1, 10, 100, 1000, 10 000, and 100 000, which allowed us to control the extent to which the raster cells crossed by a fence were more resistant to dispersal than cells not crossed by a fence. The level of significance of the estimated *Q* value was again tested using a one-tail test based on its comparison with the null distribution of *Q* values obtained from 200 stochastic rotations of dispersal vectors around their origin. In this test, the null hypothesis is that the correlation between dispersal durations and environmental distances is not higher than the correlation values estimated under such a null dispersal model where the location of installed fences had no particular impact on the wavefront velocity. The *p*-value was thus calculated as the proportion of simulated *Q* statistics being higher than the observed *Q* statistic. When the *p*-value was < 0.05, it indicates dispersal durations are significantly more positively correlated with environmental distances than with geographical distance, suggesting that the fences significantly slowed down the wavefront velocity.

For the two analyses detailed above, we used the *seraphim* package in R and the *Circuitscape* package in Julia software [18, 20].

To assess the sensitivity of our results to the assumed date of fence installation, we also ran the analyses with alternative fence installation dates defined as the start or end date of their installation period.

## Results

### Wild boar-mediated transmission clusters and wavefront cases

**Figure 3:**
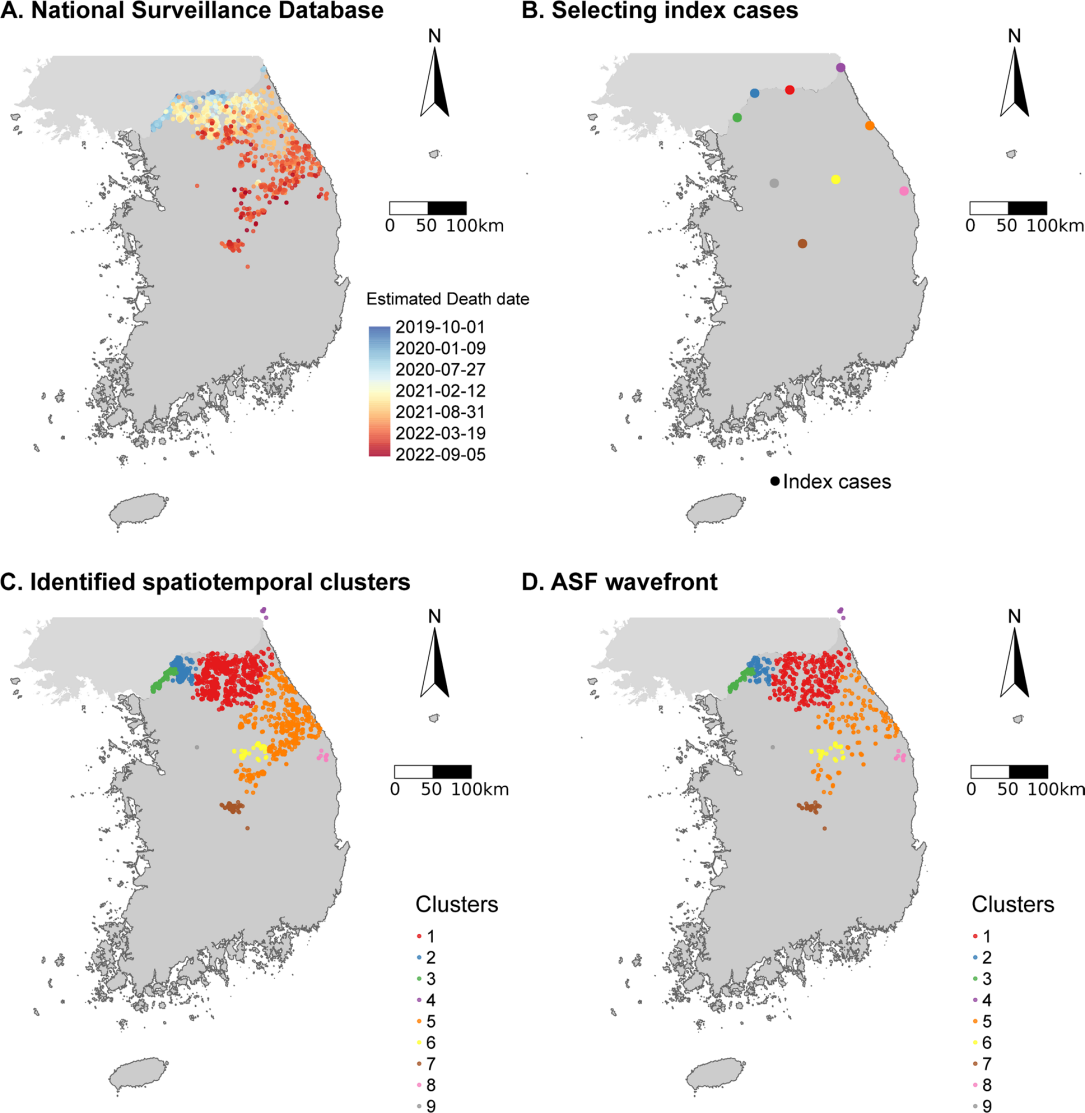
**Workflow for analysing national surveillance data.** From data collection (A) to selecting index case (B), identifying spatiotemporal clusters (C) and filtering the ASF wavefront cases (D).

**Figure 4:**
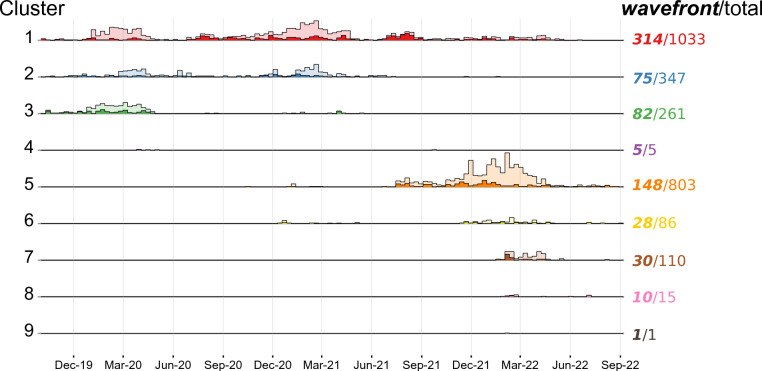
**Time series of reported and wavefront cases in each cluster.** In each row, time series of reported cases (light shade) and identified wavefront cases (dark shade) within specific cluster were plotted. The number at the end of each row indicates the number of wavefront (in bold and italic type) and reported cases (in normal type) within that respective cluster.

Among the total of 2661 cases reported in the surveillance system (Figure 3A), nine distinct seed cases were identified (Figure 3B), each of which initiated one spatiotemporal cluster (Figure 3C). Most of the cases (91.8%, 2444/2661) were included in clusters 1, 2, 3, and 5 (Figure 4). These four major clusters included 1033, 347, 261, and 803 cases, respectively. In contrast, clusters 4, 6, 7, 8 and 9 included only 5, 86, 110, 15, and 1 case(s), respectively (Figure 4).

The wild boar cases in cluster 1 started on 26 September 2019 and then spread out over the centre of the northern region of South Korea during the entire study period (Figures 3C, 4, and Additional file 3). Following the initiation of cluster 1, the cases in clusters 2 and 3 both started on 30 September 2019, and lasted up to 20 January 2022 and 10 May 2021, respectively (Figure 4, Additional file 3). They both remained very localized in the northwest part of the country (Figure 3C). The cases in cluster 5 started much later (20 October 2020) and lasted until near the end of the study period (26 August 2022). These cases were distributed over a wide area spanning from the northeast region to the central part of the country (Figures 3C, 4, and Additional file 3). The cases in cluster 4 were isolated in the northeastern part of the country and occurred in the early phases of the epizootic (27 March 2020), while the remaining clusters (6, 7, 8, and 9) occurred in the more central part of the country during the late stages of the study period (Figures 3C, 4, and Additional file 3). Cluster 9 consisted of only one human-mediated translocated case, without further wild boar-mediated transmission (Figures 3C, 4 and Additional file 3).

About a quarter of cases (26.0%, 692/2661) were considered to be wavefront cases (Figures 3D, 4 and Additional file 3). Most of the wavefront cases (89.5%, 619/692) were from the four major clusters (Figures 3D, 4 and Additional file 3). Consequently, the subsequent analyses were only applied to these four major clusters.

### Estimates of the wavefront velocity

**Figure 5:**
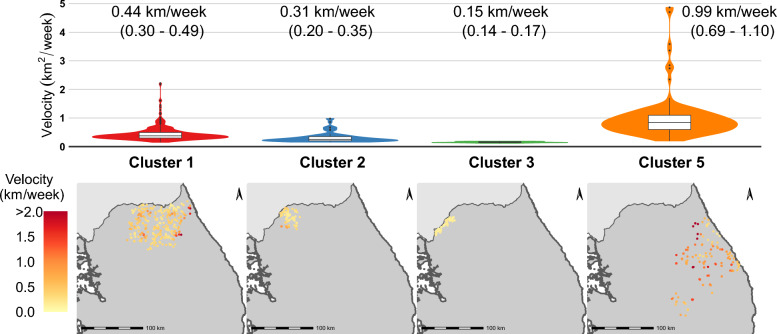
**Distribution of estimated wavefront velocities for each cluster (top) and the spatial distribution of wavefront cases for their respective clusters (bottom)**.

**Figure 6:**
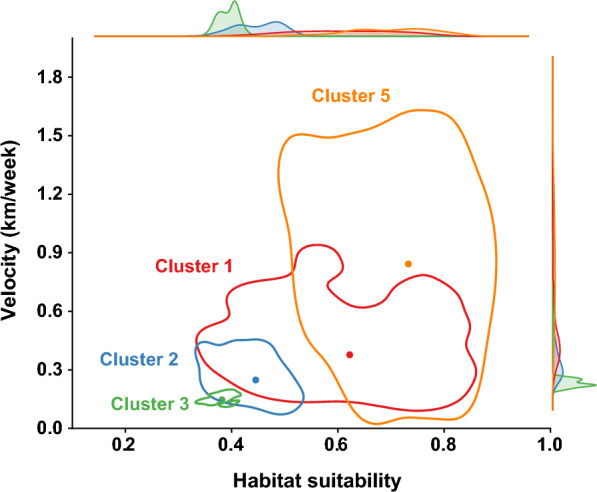
**The joint distribution of the estimated velocity as a function of wild boar habitat suitability in each cluster.** Each polygon represents the joint distribution of velocity and habitat suitability, with dots indicating the median point of velocity and habitat suitability. Marginal distributions of each variable are plotted in the margins of the plot.

Accounting for all wavefront cases, the average velocity of the ASF wavefront over four major clusters was estimated as 0.52 km/week (interquartile range [IQR]: 0.25–0.62 km/week). With an average velocity of 0.99 km/week (IQR:0.69–1.10 km/week), the virus spread faster in cluster 5 than in any of the other major clusters (average velocity of 0.44 km/week, 0.31 km/week, and 0.15 km/week for clusters 1, 2, 3, respectively, Figure 5). Joint distribution of habitat suitability and velocity for each cluster was plotted in Figure 6. This plot suggests that areas with higher habitat suitability could be associated with faster wavefronts (Figure 6).

### Impact of fencing on ASF wavefront

At the national level, fences did not show any statistically significant effect on the dispersal of ASF wavefront (Additional file 5; *N* = 1570, *p*-value = 1). However, as shown in Additional file 5, the *N* statistics analysis showed variable results at the cluster level. While the fences did not exhibit a statistically significant effect on blocking the ASF wavefront in clusters 1 (*N* = 1371, *p* = 1) and 2 (*N* = 54, *p* = 1), the results were found statistically significant in clusters 3 (*N* = 0, *p* < 0.01) and 5 (*N* = 147, *p* < 0.01) (Additional file 5).

We did not find any statistical evidence that fences impacted wavefront velocity at either the national level or the cluster level. Although national-level *Q* statistics showed a positive value with 0.014 only when *k* = 1 suggesting a reduced wavefront velocity by fencing (Additional file 4A), the impact was not found significant (Additional file 6, *p*-value = 0.46). A similar trend was obtained in one cluster (cluster 3) with a *Q* statistic of 0.018 when *k* = 1, *p*-value was above 0.05 (*p*-value = 0.46) (Additional file 6). The other clusters showed negative values of *Q* statistics under the *k* values used (Additional file 4B), suggesting that the fences did not significantly impact the wavefront velocity.

### Sensitivity analyses

The detailed results of the different sensitivity analyses are presented in Additional file 7. Changing the threshold to select the index cases for each cluster to 20 km and 40 km, expectedly changed the number of the major clusters to five and three, respectively. However, the overall wavefront velocity estimated for each alternative scenario did not differ from the baseline scenario. Most of the results based on the *N* and *Q* statistics remained consistent for all threshold distances. However, only the result from the test based on the *Q* statistic at the 20 km threshold became statistically significant for the cluster of cases that occurred at northwest regions bordering North Korea during the early phases of the epidemic. This suggests that the definition of the threshold had an influence on our conclusion regarding the impact of the fences on the ASF wavefront velocity in these northwest regions, while it had no effect on other regions. When applying a systematic change to the estimated date of death, the wavefront velocity and the results based on the *N* and *Q* statistics did not change substantially between the four distinct scenarios (see Additional file 7). Likewise, the uncertainty on the fence installation time did not change our results based on the *N* or *Q* statistics (see Additional file 7).

## Discussion

In this study, we estimated wild boar-mediated ASF wavefront dynamics, and unravelled the impact of fencing on these dynamics in South Korea, using nationwide surveillance data. The average estimated velocity of the ASF wavefront at the national level was 0.52 km/week, and ranged from 0.69 to 1.10 km/week depending on the spatiotemporal clusters. Fencing was shown to have a significant impact on ASF virus dispersal in two of the four main clusters of ASF cases in wild boar. This result was robust to potential deviations from initial model assumptions. We found no evidence that the fences significantly affected the wavefront velocity, except in the northwest regions, where the outcomes were dependent on the assumption made to define the clusters of wild boar-mediated cases. Consequently, given the data available, we can confidently conclude on this aspect only for areas outside of these northwest regions.

We estimated the overall ASF wavefront velocity as 0.52 km/week, which is faster but still comparable to what was estimated for the 2018–2019 epizootic that occurred in Belgium (0.39 km/week) [10]. The model assumptions are unlikely to have induced this discrepancy, as verified by the sensitivity analysis. However, the disparate wild boar densities between the two countries could explain this discrepancy in the velocities of the ASF wavefront. Specifically, the wild boar density in some ASF-affected regions in South Korea exceeded 6 wild boar/km^2^, notably higher than 3.12 wild boar/km^2^ in Belgium [12, 21].

Our findings highlighted the regional heterogeneity of the velocity, with a higher velocity in the eastern region (cluster 5) than in the northwest (clusters 2 and 3). This heterogeneity across the clusters could be attributed to the habitat suitability of wild boar in South Korea [5, 17]. The natural landscape in the eastern region of the country remains well preserved, featuring predominantly forests and mountains, which favour wild boar habitat, activity and movement [22], while the northwest region is much more urbanized. The eastern region is therefore much more suitable for wild boar [17], favouring greater densities and higher contact rates and thus promoting ASF dispersal. Thus, as illustrated in Figure 6, there is a high likelihood that the eastern side of the Korean peninsula is acting as a highway for the ASF wavefront. Further empirical evidence is needed to substantiate this association.

In Belgium, Dellicour et al. formally demonstrated that the network of fences impacted both ASF dispersal and wavefront velocity during the 2018–2019 epizootic [10]. In the context of South Korea, using the same methodological approaches, we found that fencing was much less impactful than in Belgium. The analysis at the cluster level revealed an effect of the fences on the dispersal of ASF only for clusters 3 and 5, while no effects on the wavefront velocity were found for any of the clusters. The effectiveness of control measures for ASF in wild boar populations is contingent on several spatial and temporal parameters, including the speed at which the wavefront spreads and the location of fence installation [7, 23–26]. If ASFV spreads quickly and has already traversed a certain area where fences were installed, the wavefront can still progress beyond the fences, negating the impact of the fences on the wavefront. Thus, a fence should be placed at an adequate distance from the detected wavefront with consideration of the wavefront velocity to effectively impede the disease’s dynamics. In the case of cluster 5, the corresponding fences were installed at further distances from the detected wavefront compared to the distances of the fences built in cluster 1 (Additional file 1B, and Additional file 8), potentially resulting in a greater impact of fencing in cluster 5. In clusters 1 and 2, undetected ASF cases likely passed through the areas where fences were still under construction and not yet completed, explaining the weakness of the defence despite the high density of fences. In cluster 3, where the wavefront progressed at a slower pace, the installed fences managed to catch up and impacted the dispersal. To ensure the optimal placement of fences, it is crucial to consider that ASF epidemics in wild boar is only partially observed. Moreover, the effectiveness of fencing is assessed to be coupled with other measures, including carcass search, and depopulation [9]. However, accounting for these factors to plan interventions does not come without important methodological challenges. Mechanistic models of ASF transmission, as reviewed in [27], can offer a promising approach for optimizing the control measures.

Other factors were suggested as factors to influence the effectiveness of fencing, including the type of fence and their maintenance. Previous study indicated the big-game-proof type of fences, being 200 cm tall with tightened horizontal and vertical wires (15 × 15 cm), were more effective than the livestock type of fences, which stand at 130 cm and have wider gaps between the wires (30 cm wide between vertical lines) [28]. The South Korean government has installed two different kinds of wild boar-proof fences [29]. Unfortunately, we did not have detailed information on the type of fences used in South Korea, preventing us from assessing their effectiveness. Heavy rain and resulting ground collapses can also damage the installed fences, which was suggested as a potential weak spot in the strategy to control ASF in wild boar in South Korea [30]. In other contexts, watercourses have been shown to weaken the effectiveness of fencing by serving as crossing hotspots [28, 31, 32]. Moreover, the age and sex of the wild boar may modify the effectiveness of fences as male and young wild boar tend to behave actively and roam across wider areas. These two factors can shape the effectiveness of fences during mating seasons as well. It is imperative to identify and understand the interplay between these various potential factors affecting the effectiveness of fencing. This knowledge can optimize fencing strategies for more effective ASF management in wild boar populations.

Finally, it is essential to discuss the impact of two important limitations of the data, including the estimated date of death and the likely under-detection of wild boar cases. While the death date of ASF-positive carcasses was estimated by ME experts, we acknowledge that these estimates might be uncertain, which could bias the estimations of the velocity and the effectiveness of fencing. The post-mortem decomposition process is affected greatly by several factors, such as rainfall, daily temperature, humidity, sunlight, and scavengers. When a carcass has been in the environment for a long time, these factors interact with each other, further increasing the potential for uncertainty in the estimation [33]. These factors have a particularly dramatic effect on post-mortem processes during summer in both the South Korean context [34] and European context [35]. Because the effect of these factors accumulates over time, the longer the delay between death and carcass detection, the more uncertain the estimated date of death. However, the majority of the cases (95% and 50%) were estimated to be reported less than 45 days and 7 days, respectively, after their estimated date of death. During these time frames, wild boar carcasses are less likely to exhibit a dramatic change in decomposition, thus leading to a lower uncertainty in the estimate of the death date [35]. Moreover, in South Korea, only a few small scavengers remain, including raccoon dogs (*Nyctereutes procyonoides*) and ravens (*Corvus corone*) [5], so their activity is less likely to affect decomposition rates. Also, the sensitivity analysis supports the minimal impact of the uncertainty in estimated date of death on our findings. Therefore, we argue that the potential bias derived from the uncertainty of the death date had a limited impact on our conclusions. For a more robust understanding of ASF disease dynamics in wild boar, it is crucial to conduct further research on post-mortem decomposition specific to wild boar.

Despite the implementation of intensive surveillance in wild boar, the number of detected ASF-positive wild boar only represented a fraction of the true number of infected wild boar [12, 36, 37]. This under-detection might impact the wavefront velocity estimates and the assessment of the effectiveness of fencing. However, given that a zoning approach for ASF surveillance in wild boar has been implemented around reported cases [38], we consider that under-detection had a limited impact on the estimated wavefront velocity. Moreover, as a part of the velocity estimations, we spatially interpolated the estimated date of death, which could reduce the bias derived from the under-detection. Lastly, adopting the approach of Dellicour et al. [10], we represented the wild boar-mediated transmission chain as a simplified straight-line proxy when calculating *N* statistic. This dispersal model was designed to emulate the surveillance efforts, by maintaining the same distribution of geographic distances between the index case and subsequent cases that extend the infected area in each cluster. By comparing the observed case data with randomized datasets with equivalent sampling efforts, we mitigated the impact of the sampling bias. However, we acknowledge that it could reduce the statistical power of our analysis, which may suggest that the significant results identified from the *N* and *Q* statistics are substantial.

Our findings on wavefront velocity and effectiveness of fencing suggest that the location of fencing and the ASF wavefront dynamics intertwined with the effectiveness of fencing, emphasizing the need to better understand the latent spread of disease diffusion. More precisely, there is a need to better estimate the time it takes between the invasion of a location and the first carcass detection in order to install fences in optimal spatiotemporal locations. Our results also emphasise the critical importance of understanding accurately the local epidemiological context and of having a precise estimation of the disease distribution.

## Supplementary Information

**Additional file 1:** **Fence construction and the spatial distribution of ASF-positive wild boar cases**.

**Additional file 2:** **Estimation of reporting delay of ASF-positive wild boar carcass.**

**Additional file 3:** **Period, cases and selected wavefront cases in each cluster.**

**Additional file 4:** **Results of Q statistic and regression coefficient for different k values.**

**Additional file 5:** **Results of statistical significance test of N statistics.**

**Additional file 6:** **Results of statistical significance test of Q statistics.**

**Additional file 7:** **Sensitivity analysis under several assumptions for spread rate analysis, N and Q statistics.**

**Additional file 8:** **The distribution of selected wavefront cases in each cluster and installed fencing.**

## Data Availability

The datasets used and/or analysed during the current study are available from the corresponding author on reasonable request.

## References

[1] World Organisation for Animal Health (2023) WOAH-Listed diseases. https://www.woah.org/en/what-we-do/animal-health-and-welfare/animal-diseases/. Accessed 20 Sep 2023

[2] Chenais E, Stahl K, Guberti V, Depner K (2018) Identification of wild boar-habitat epidemiologic cycle in African swine fever epizootic. Emerg Infect Dis 24:810–812 29553337 10.3201/eid2404.172127 PMC5875284

[3] Dixon LK, Stahl K, Jori F, Vial L, Pfeiffer DU (2020) African swine fever epidemiology and control. Annu Rev Anim Biosci 8:221–246 31743062 10.1146/annurev-animal-021419-083741

[4] Sanchez-Vizcaino JM, Mur L, Gomez-Villamandos JC, Carrasco L (2015) An update on the epidemiology and pathology of African swine fever. J Comp Pathol 152:9–21 25443146 10.1016/j.jcpa.2014.09.003

[5] Lim J-S, Andraud M, Kim E, Vergne T (2023) Three years of African swine fever in South Korea (2019–2021): a scoping review of epidemiological understanding. Transbound Emerg Dis 2023:4686980 10.1155/2023/4686980 PMC12016690 40303829

[6] Ministry of Environments (2021) Status of wild boar ASF (African swine fever) outbreak in Korea. https://www.mafra.go.kr/FMD-AI2/map/ASF/ASF_map.jsp. Accessed 20 Sep 2022

[7] European Food Safety Authority, Boklund A, Cay B, Depner K, Foldi Z, Guberti V, Masiulis M, Miteva A, More S, Olsevskis E, Satran P, Spiridon M, Stahl K, Thulke HH, Viltrop A, Wozniakowski G, Broglia A, Cortinas Abrahantes J, Dhollander S, Gogin A, Verdonck F, Amato L, Papanikolaou A, Gortazar C (2018) Epidemiological analyses of African swine fever in the European Union (November 2017 until November 2018). EFSA J 16:5494 10.2903/j.efsa.2018.5494 PMC7009685 32625771

[8] Han JH, Yoo DS, Pak SI, Kim ET (2021) Understanding the transmission of African swine fever in wild boars of South Korea: a simulation study for parameter estimation. Transbound Emerg Dis 69:e1101–e1112 34821474 10.1111/tbed.14403

[9] Lange M, Guberti V, Thulke HH (2018) Understanding ASF spread and emergency control concepts in wild boar populations using individual‐based modelling and spatio‐temporal surveillance data. EFSA Support Publ 15:EN-1521

[10] Dellicour S, Desmecht D, Paternostre J, Malengreaux C, Licoppe A, Gilbert M, Linden A, Park A (2020) Unravelling the dispersal dynamics and ecological drivers of the African swine fever outbreak in Belgium. J Appl Ecol 57:1619–1629

[11] Ministry of Environment Republic of Korea (2022) Press releass from Ministry of Environment Republic of Korea. https://27.101.216.208/home/web/board/list.do?menuId=10525&boardMasterId=1&boardCategoryId=39. Accessed 24 Oct 2023

[12] Jo YS, Gortázar C (2021) African swine fever in wild boar: assessing interventions in South Korea. Transbound Emerg Dis 68:2878–2889 33844467 10.1111/tbed.14106

[13] Duong T (2007) ks: Kernel density estimation and kernel discriminant analysis for multivariate data in R. J Stat Softw 21:1–16

[14] Tisseuil C, Gryspeirt A, Lancelot R, Pioz M, Liebhold A, Gilbert M (2016) Evaluating methods to quantify spatial variation in the velocity of biological invasions. Ecography 39:409–418

[15] Kraemer MUG, Reiner RC Jr, Brady OJ, Messina JP, Gilbert M, Pigott DM, Yi D, Johnson K, Earl L, Marczak LB, Shirude S, Davis Weaver N, Bisanzio D, Perkins TA, Lai S, Lu X, Jones P, Coelho GE, Carvalho RG, Van Bortel W, Marsboom C, Hendrickx G, Schaffner F, Moore CG, Nax HH, Bengtsson L, Wetter E, Tatem AJ, Brownstein JS et al (2019) Past and future spread of the arbovirus vectors *Aedes aegypti* and *Aedes albopictus*. Nat Microbiol 4:854–863 30833735 10.1038/s41564-019-0376-y PMC6522366

[16] Nychka D, Furrer R, Paige J, Sain S, Nychka MD (2015) Package ‘fields’: tools for spatial data. R package version 16.3. https://cran.r-project.org/web/packages/fields/index.html. Accessed 20 Oct 2022

[17] Kim E-T, Pak S-I (2020) Species distribution modeling for wild boar (*Sus scropa*) in the Republic of Korea using MODIS data. J Prev Vet Med 44:89–95

[18] Anantharaman R, Hall K, Shah V, Edelman A (2019) Circuitscape in Julia: High performance connectivity modelling to support conservation decisions. arXiv preprint arXiv:1906.03542

[19] McRae BH (2006) Isolation by resistance. Evolution 60:1551–1561 17017056

[20] Dellicour S, Rose R, Faria NR, Lemey P, Pybus OG (2016) SERAPHIM: studying environmental rasters and phylogenetically informed movements. Bioinformatics 32:3204–3206 27334476 10.1093/bioinformatics/btw384

[21] Licoppe A, De Waele V, Malengreaux C, Paternostre J, Van Goethem A, Desmecht D, Herman M, Linden A (2023) Management of a focal introduction of ASF Virus in wild boar: the Belgian experience. Pathogens 12:152 36839424 10.3390/pathogens12020152 PMC9961158

[22] Ballari SA, Barrios-García MN (2014) A review of wild boar *Sus scrofa* diet and factors affecting food selection in native and introduced ranges. Mamm Rev 44:124–134

[23] Reichold A, Lange M, Thulke HH (2022) Modelling the effectiveness of measures applied in zones dedicated to stop the spread of African Swine Fever in wild boar when bordering with a region of limited control. EFSA Support Publ 19:EN-7320

[24] Lange M, Reichold A, Thulke HH (2021) Modelling wild boar management for controlling the spread of ASF in the areas called white zones (zones blanche). EFSA Support Publ 18:EN-6573

[25] European Food Safety Authority, Banos JV, Boklund A, Gogin A, Gortazar C, Guberti V, Helyes G, Kantere M, Korytarova D, Linden A, Masiulis M, Miteva A, Neghirla I, Olsevskis E, Ostojic S, Petr S, Staubach C, Thulke HH, Viltrop A, Wozniakowski G, Broglia A, Abrahantes Cortinas J, Dhollander S, Mur L, Papanikolaou A, Van der Stede Y, Zancanaro G, Stahl K (2022) Epidemiological analyses of African swine fever in the European Union: (September 2020 to August 2021). EFSA J 20:7290 10.2903/j.efsa.2022.7290 PMC9066549 35515335

[26] European Food Safety Authority, Desmecht D, Gerbier G, Gortazar Schmidt C, Grigaliuniene V, Helyes G, Kantere M, Korytarova D, Linden A, Miteva A, Neghirla I, Olsevskis E, Ostojic S, Petit T, Staubach C, Thulke HH, Viltrop A, Richard W, Wozniakowski G, Cortinas JA, Broglia A, Dhollander S, Lima E, Papanikolaou A, Van der Stede Y, Stahl K (2021) Epidemiological analysis of African swine fever in the European Union (September 2019 to August 2020). EFSA J 19:6572 10.2903/j.efsa.2021.6572 PMC8100952 33976715

[27] Hayes BH, Andraud M, Salazar LG, Rose N, Vergne T (2021) Mechanistic modelling of African swine fever: a systematic review. Prev Vet Med 191:105358 33930624 10.1016/j.prevetmed.2021.105358

[28] Laguna E, Barasona JA, Carpio AJ, Vicente J, Acevedo P (2022) Permeability of artificial barriers (fences) for wild boar (*Sus scrofa*) in Mediterranean mixed landscapes. Pest Manag Sci 78:2277–2286 35229454 10.1002/ps.6853 PMC9313896

[29] Minstry of Environment (2019) Enhanced control measures for African Swine Fever in wild boars implemented. https://www.mafra.go.kr/bbs/mafra/68/321809/artclView.do. Accessed 2 Oct 2023

[30] Ito S, Bosch J, Jeong H, Aguilar-Vega C, Park J, Martinez-Aviles M, Sanchez-Vizcaino JM (2022) Spatio-temporal epidemiology of the spread of African swine fever in wild boar and the role of environmental factors in South Korea. Viruses 14:2779 36560783 10.3390/v14122779 PMC9782897

[31] Cozzi G, Broekhuis F, McNutt JW, Schmid B (2013) Comparison of the effects of artificial and natural barriers on large African carnivores: implications for interspecific relationships and connectivity. J Anim Ecol 82:707–715 23402594 10.1111/1365-2656.12039

[32] Honda T (2019) A sound deterrent prevented deer intrusions at the intersection of a river and fence. Mammal Study 44:269–274

[33] Brooks JW (2016) Postmortem changes in animal carcasses and estimation of the postmortem interval. Vet Pathol 53:929–940 26945004 10.1177/0300985816629720

[34] Cho H-K, Kim E-T, Jung B-S, Pak S-I (2021) A preliminary investigation into the decomposition rate of wild boar carcasses in forest habitats. J Prev Vet Med 45:44–52

[35] Probst C, Gethmann J, Amendt J, Lutz L, Teifke JP, Conraths FJ (2020) Estimating the postmortem interval of wild boar carcasses. Vet Sci 7:6 31948042 10.3390/vetsci7010006 PMC7157510

[36] Lim J-S, Vergne T, Pak SI, Kim E (2021) Modelling the spatial distribution of ASF-positive wild boar carcasses in South Korea Using 2019–2020 National Surveillance Data. Animals 11:1208 33922261 10.3390/ani11051208 PMC8145688

[37] Jo YS, Gortazar C (2020) African swine fever in wild boar, South Korea, 2019. Transbound Emerg Dis 67:1776–1780 32145154 10.1111/tbed.13532

[38] Kim YJ, Park B, Kang HE (2021) Control measures to African swine fever outbreak: active response in South Korea, preparation for the future, and cooperation. J Vet Sci 22:e13 33522165 10.4142/jvs.2021.22.e13 PMC7850787

[39] Akhmetzhanov AR, Jung SM, Cheng HY, Thompson RN (2021) A hospital-related outbreak of SARS-CoV-2 associated with variant Epsilon (B.1.429) in Taiwan: transmission potential and outbreak containment under intensified contact tracing, January-February 2021. Int J Infect Dis 110:15–20 34146689 10.1016/j.ijid.2021.06.028 PMC8214728

[40] Stan Model for Report Delay https://github.com/borizook/reporting_delay_ASF. Accessed 14 Nov 2022

[41] Stan Development Team (2023) RStan: the R interface to Stan

[42] Gelman A, Carlin JB, Stern HS, Dunson DB, Vehtari A, Rubin DB (2013) Bayesian data analysis. CRC Press, Boca Raton

