# Supplementary material for: A spatially-heterogeneous impact of fencing on the African swine fever wavefront in the Korean wild boar population
Source: Vet Res. 2024 Dec 18;55:163. doi: 10.1186/s13567-024-01422-7 (PMC11654197; doi:10.1186/s13567-024-01422-7)
Supplement: Supplementary file 1 — Additional file 1: Fence construction and the spatial distribution of ASF-positive wild boar cases. [file 13567_2024_1422_MOESM1_ESM.docx]

**Additional file 1**


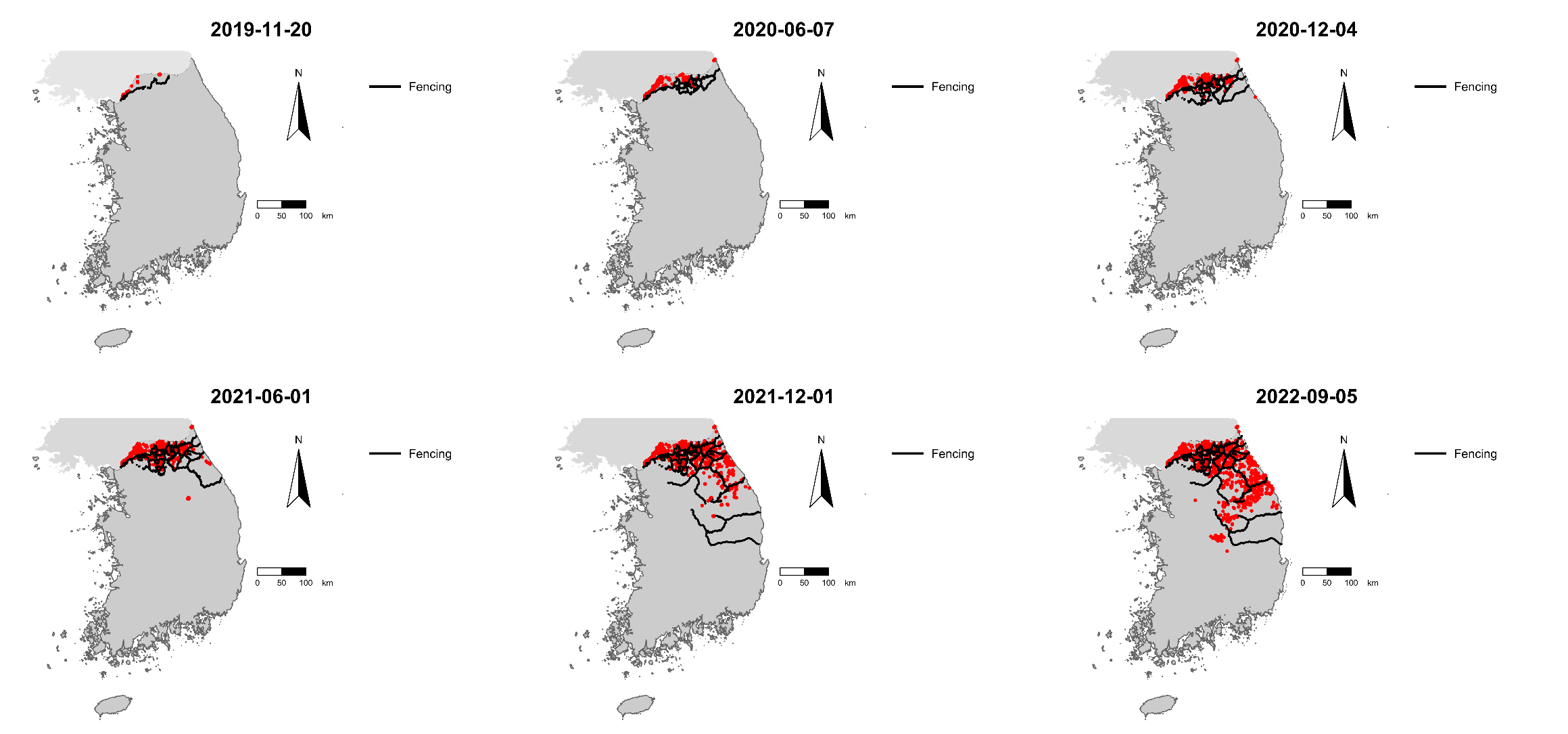


**A. Timing of the fence construction and the spatial distribution of ASF-positive wild boar cases**


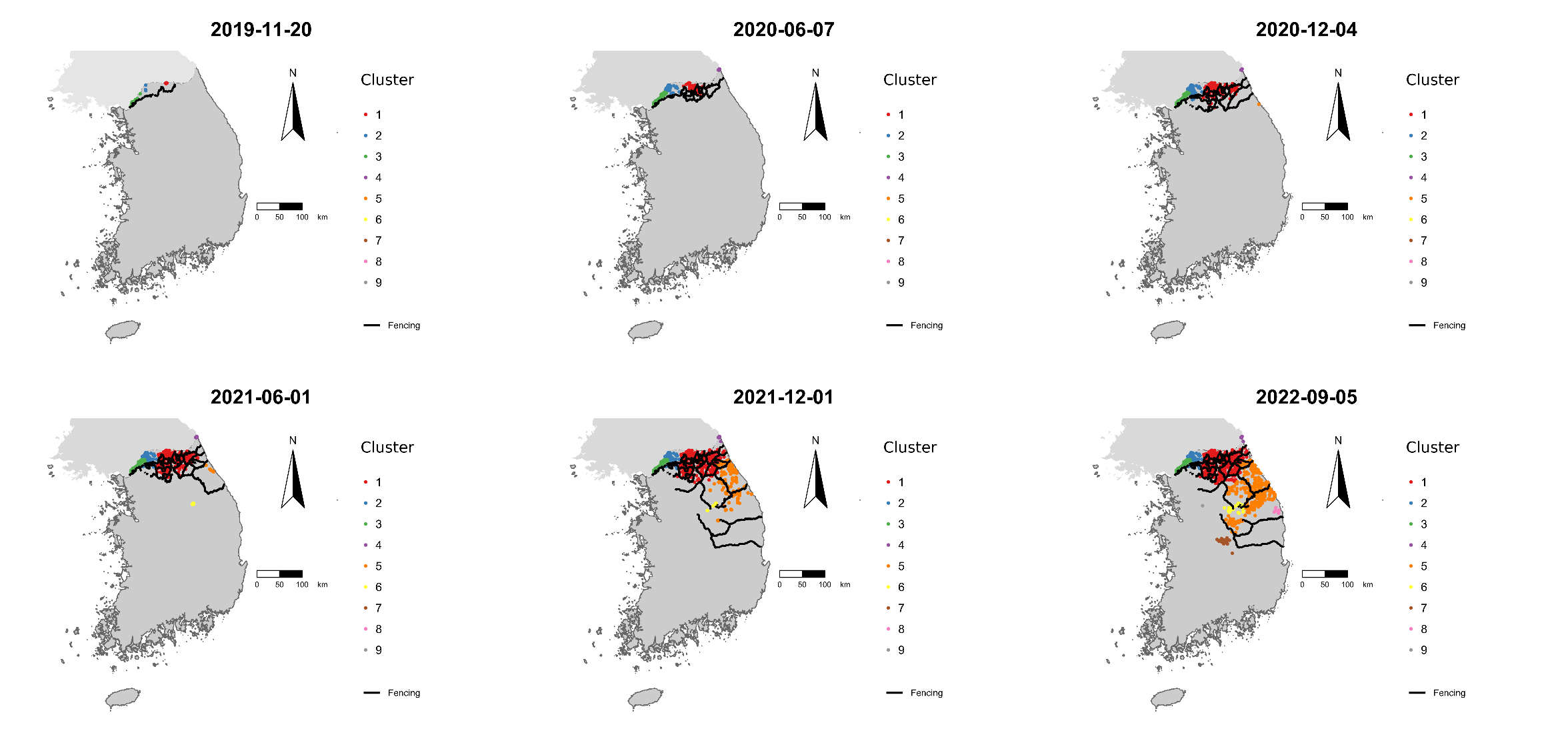


**B. Timing of the fence construction and the spatial distribution of ASF-positive wild boar cases in each cluster.** Each coloured dot indicates the ASF-positive wild boar cases in corresponding cluster.
