# Supplementary material for: A spatially-heterogeneous impact of fencing on the African swine fever wavefront in the Korean wild boar population
Source: Vet Res. 2024 Dec 18;55:163. doi: 10.1186/s13567-024-01422-7 (PMC11654197; doi:10.1186/s13567-024-01422-7)
Supplement: Supplementary file 2 — Additional file 2: Estimation of reporting delay of ASF-positive wild boar carcass. [file 13567_2024_1422_MOESM2_ESM.docx]

**Additional file 2 Estimation of reporting delay of ASF-positive wild boar carcass**

**Methods**

The analysis focused on the estimation of reporting delay, defined as the time difference between the detection date of wild boar carcasses and their estimated death date. Specifically, this analysis was confined to ASF-positive carcass samples. Out of a total of 2661 wild boar cases, 2384 cases from carcass samples were included, excluding carcasses with zero reporting delay (five cases) and those with unidentifiable death dates (six cases).

The reporting delay was estimated within a Bayesian framework by modeling it as a mixture of three probability distributions: gamma, Weibull, and lognormal. This approach allowed for capturing the variability in reporting delay using a combination of these distribution types. The likelihood function integrated three component likelihoods, each weighted according to its significance.

The code for this analysis was adapted from a previous study by Akhmetzhanov, Jung, Cheng and Thompson [40] and was further customized to suit the specific requirements of our analysis. The code can be found in this link [41]. The analysis was conducted using the *rstan* package within the R software environment [42].

We conducted a Markov chain Monte Carlo (MCMC) sampling with four chains, each consisting of 20 000 iterations. To ensure the stability of the results, we applied a burn-in of 7000 iterations to remove the initial transient behavior of the chains. Convergence of the chains was assessed through visual inspection of trace plots and Gelman–Rubin–Brooks diagnostic [43].

The posterior distribution of model parameters was summarized using the median and the 95% credible intervals (95% CrI).

**Results**

The mean reporting delay obtained from the MCMC analysis was calculated to be 13.8 days, with a 95% credible interval ranging from 13.2 to 14.5 days. Additionally, the standard deviation of the reporting delay was determined to be 13.3 days, within a 95% credible interval of 12.5 to 14.2 days (Figure 1). The chains showed appropriate converged in visual inspection and Gelman–Rubin–Brooks diagnostic showed under 1.01, indicating the appropriate convergence.


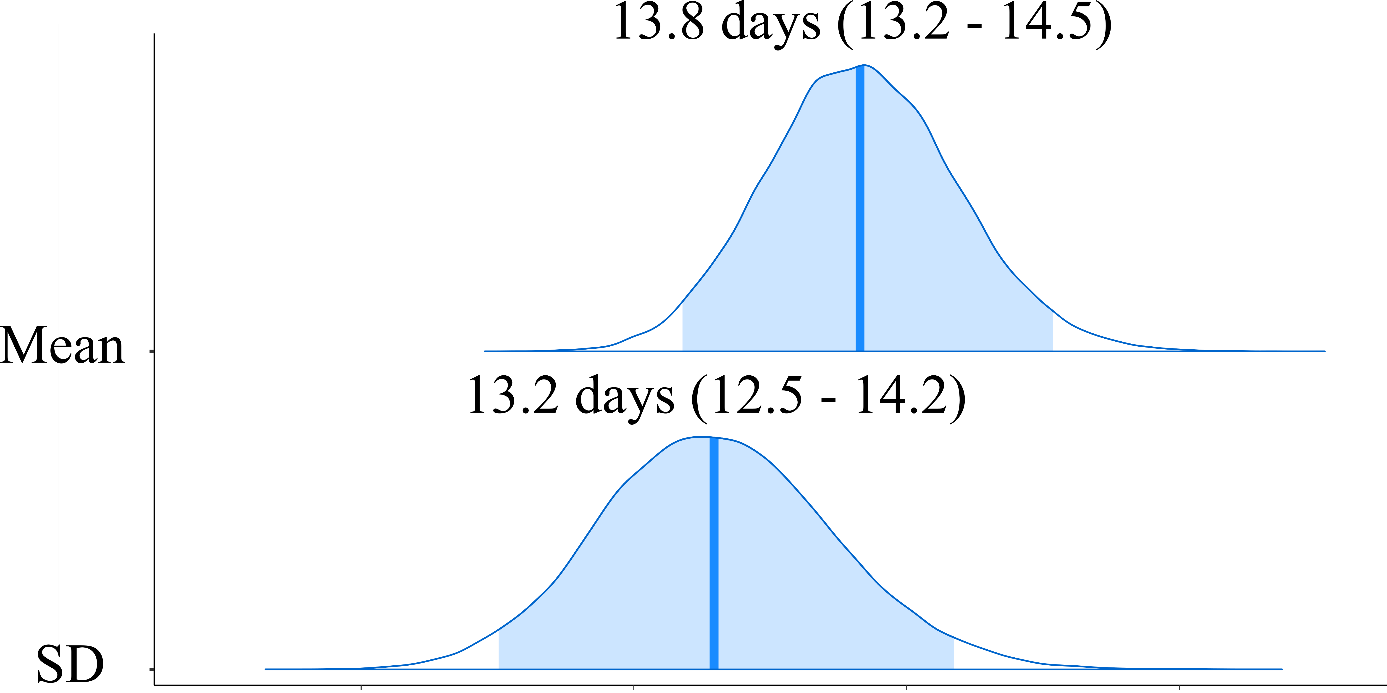


**Figure 1. Density of posterior distributions of mean and standard deviation of the reporting delay.** Vertical lines indicate the median value of each distribution and the lightly colored area with each distirbution represents the 95% credible interval.
