# Supplementary material for: A spatially-heterogeneous impact of fencing on the African swine fever wavefront in the Korean wild boar population
Source: Vet Res. 2024 Dec 18;55:163. doi: 10.1186/s13567-024-01422-7 (PMC11654197; doi:10.1186/s13567-024-01422-7)
Supplement: Supplementary file 3 — Additional file 3: Period, cases and selected wavefront cases in each cluster. [file 13567_2024_1422_MOESM3_ESM.docx]

**Additional file 3 Period, cases and selected wavefront cases in each cluster**

| **Cluster** | **Disease dynamics** | **Start** | **End** | **Cases** | **^*^Wavefront cases** |
| --- | --- | --- | --- | --- | --- |
| 1 | Observed | 2019-10-11 | 2022-08-17 | 1033 | 314 |
|  | Underlying | 2019-09-26 | 2022-06-18 |  |  |
| 2 | Observed | 2019-10-02 | 2022-03-01 | 347 | 75 |
|  | Underlying | 2019-09-30 | 2022-01-20 |  |  |
| 3 | Observed | 2019-10-11 | 2021-05-15 | 261 | 82 |
|  | Underlying | 2019-09-30 | 2021-05-10 |  |  |
| 4 | Observed | 2020-03-31 | 2021-09-24 | 5 | 5 |
|  | Underlying | 2020-03-27 | 2021-09-24 |  |  |
| 5 | Observed | 2021-01-04 | 2022-09-15 | 803 | 148 |
|  | Underlying | 2020-10-20 | 2022-08-26 |  |  |
| 6 | Observed | 2020-12-28 | 2022-09-10 | 86 | 28 |
|  | Underlying | 2020-12-12 | 2022-09-08 |  |  |
| 7 | Observed | 2022-01-23 | 2022-08-04 | 11 | 30 |
|  | Underlying | 2022-01-24 | 2022-07-04 |  |  |
| 8 | Observed | 2022-02-08 | 2022-07-04 | 15 | 10 |
|  | Underlying | 2022-01-23 | 2022-08-04 |  |  |
| 9 | Observed | 2022-03-11 | 2022-03-11 | 1 | 1 |
|  | Underlying | 2022-02-09 | 2022-02-09 |  |  |

^*^ ASF wavefront cases were defined as the ASF cases that were have died, been hunted, or trapped at time *t+1* outside the ASF-affected area. This area was delineated by 95% kernel density polygon constructed from the all cases that have died, been hunted, or trapped at time *t*.
