# Supplementary material for: A spatially-heterogeneous impact of fencing on the African swine fever wavefront in the Korean wild boar population
Source: Vet Res. 2024 Dec 18;55:163. doi: 10.1186/s13567-024-01422-7 (PMC11654197; doi:10.1186/s13567-024-01422-7)
Supplement: Supplementary file 4 — Additional file 4: Results of Q statistic and regression coefficient for different k values. [file 13567_2024_1422_MOESM4_ESM.docx]

**Additional file 4**

**A. National-level *Q* statistic and regression coefficient for different *k* values**

| *k* value | Regression coefficient | *Q* statistics |
| --- | --- | --- |
| 1 | 971.715 | 0.014 |
| 5 | 456.667 | -0.185 |
| 10 | 227.817 | -0.318 |
| 100 | 30.737 | -0.473 |
| 500 | 12.919 | -0.496 |
| 1000 | 1.968 | -0.517 |
| 5000 | 0.925 | -0.520 |
| 10000 | 0.173 | -0.523 |
| 50000 | 0.086 | -0.523 |
| 100000 | 0.008 | -0.523 |

**B. Cluster-level *Q* statistics and regression coefficient for different *k* values**

| **Cluster** | ***k* value** | **Regression coefficient** | ***Q* statistics** |
| --- | --- | --- | --- |
| **Cluster 1** | 1 | 992.471 | -0.080 |
|  | 5 | 338.105 | -0.370 |
|  | 10 | 134.589 | -0.476 |
|  | 100 | 9.587 | -0.552 |
|  | 500 | 2.783 | -0.558 |
|  | 1000 | 0.101 | -0.560 |
|  | 5000 | 0.011 | -0.560 |
|  | 10000 | -0.005 | -0.560 |
|  | 50000 | -0.003 | -0.560 |
|  | 100000 | 0.000 | -0.560 |
| **Cluster 2** | 1 | 899.423 | -0.001 |
|  | 5 | 359.950 | -0.145 |
|  | 10 | 181.654 | -0.228 |
|  | 100 | 29.080 | -0.333 |
|  | 500 | 13.317 | -0.351 |
|  | 1000 | 2.390 | -0.367 |
|  | 5000 | 1.174 | -0.370 |
|  | 10000 | 0.231 | -0.372 |
|  | 50000 | 0.115 | -0.372 |
|  | 100000 | 0.012 | -0.372 |
| **Cluster 3** | 1 | 810.440 | 0.018 |
|  | 5 | 513.543 | -0.068 |
|  | 10 | 250.502 | -0.165 |
|  | 100 | 16.199 | -0.260 |
|  | 500 | 4.013 | -0.265 |
|  | 1000 | 0.049 | -0.267 |
|  | 5000 | -0.027 | -0.267 |
|  | 10000 | -0.014 | -0.267 |
|  | 50000 | -0.008 | -0.267 |
|  | 100000 | -0.001 | -0.267 |
| **Cluster 5** | 1 | 541.613 | -0.011 |
|  | 5 | 380.695 | -0.117 |
|  | 10 | 214.952 | -0.231 |
|  | 100 | 14.886 | -0.370 |
|  | 500 | 3.038 | -0.378 |
|  | 1000 | -0.429 | -0.378 |
|  | 5000 | -0.332 | -0.377 |
|  | 10000 | -0.094 | -0.376 |
|  | 50000 | -0.049 | -0.375 |
|  | 100000 | -0.005 | -0.375 |
