# Supplementary material for: A spatially-heterogeneous impact of fencing on the African swine fever wavefront in the Korean wild boar population
Source: Vet Res. 2024 Dec 18;55:163. doi: 10.1186/s13567-024-01422-7 (PMC11654197; doi:10.1186/s13567-024-01422-7)
Supplement: Supplementary file 6 — Additional file 6: Results of statistical significance test of Q statistics. [file 13567_2024_1422_MOESM6_ESM.docx]

**Additional file 6 The null and observed distributions of national and cluster-level *Q* statistics.**


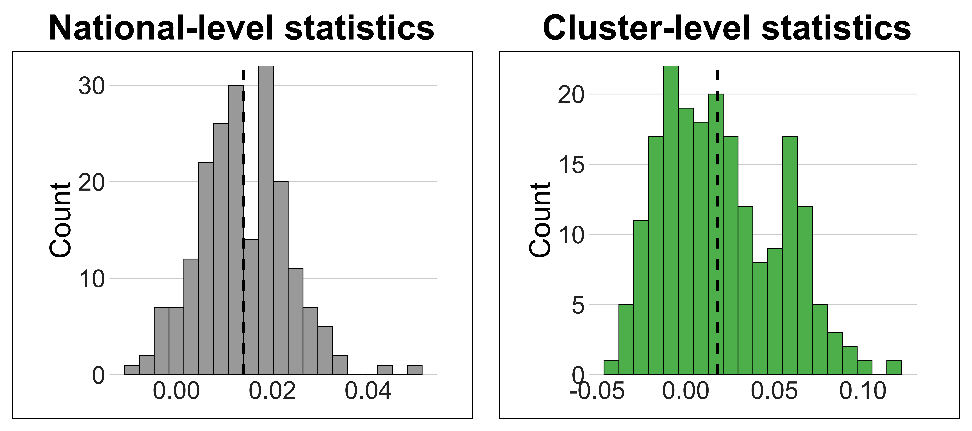


Each histogram represents null distribution of national-level ***Q*** statistics and cluster-level ***Q*** statistics for cluster 3 in order. Dashed line indicates the observed ***Q*** statistics.
