# Supplementary material for: A spatially-heterogeneous impact of fencing on the African swine fever wavefront in the Korean wild boar population
Source: Vet Res. 2024 Dec 18;55:163. doi: 10.1186/s13567-024-01422-7 (PMC11654197; doi:10.1186/s13567-024-01422-7)
Supplement: Supplementary file 7 — Additional file 7: Sensitivity analysis under several assumptions for spread rate analysis, N and Q statistics. [file 13567_2024_1422_MOESM7_ESM.docx]

**Additional file 7 Sensitivity Analyses**

Contents

1. Sensitivity analyses under varying geographical criteria
   1. 20 km of geographical distance to select the index cases
   2. 40 km of geographical distance to select the index cases
2. Sensitivity analyses under varying reporting delay
   1. 50% Shorter Reporting Delay
   2. 20% Shorter Reporting Delay
   3. 20% Longer Reporting Delay
   4. 50% Longer Reporting Delay
3. Sensitivity analyses under varying installation time of fences.
   1. Starting Date of fence installation
   2. Ending Date of fence installation
4. **Sensitivity analyses under varying geographical criteria**

**The scenarios tested in this sensitivity analyses**

1. 20 km of geographical distance to select the index cases
2. 40 km of geographical distance to select the index cases

**The outputs from the sensitivity analysis**

1. Cluster identifications
2. Wavefront velocity
3. *N* statistic
4. *Q* statistic

**Table A1. Summary of the sensitivity analysis under varying geographical criteria to select index case**

|  | Cluster | Velocity  Mean (IQR) | *N* statistics | | | *Q* statistics | |
| --- | --- | --- | --- | --- | --- | --- | --- |
|  |  |  | National level | Cluster level | National level | | Cluster level |
| Baseline | Four major clusters | 0.52  (0.25 – 0.62) | Not significant | Significant in two clusters | Not significant | | Not significant |
| 20 km | Three major clusters | 0.54  (0.29 – 0.66) | Not significant | Significant in one cluster* | Not significant | | Significant in one cluster |
| 40 km | Five major clusters | 0.50  (0.25 – 0.68) | Not significant | Significant in two clusters** | Not significant | | Not significant |

*, **: The clusters that showed significant *N* statistics geographically corresponds to those in baseline analysis.


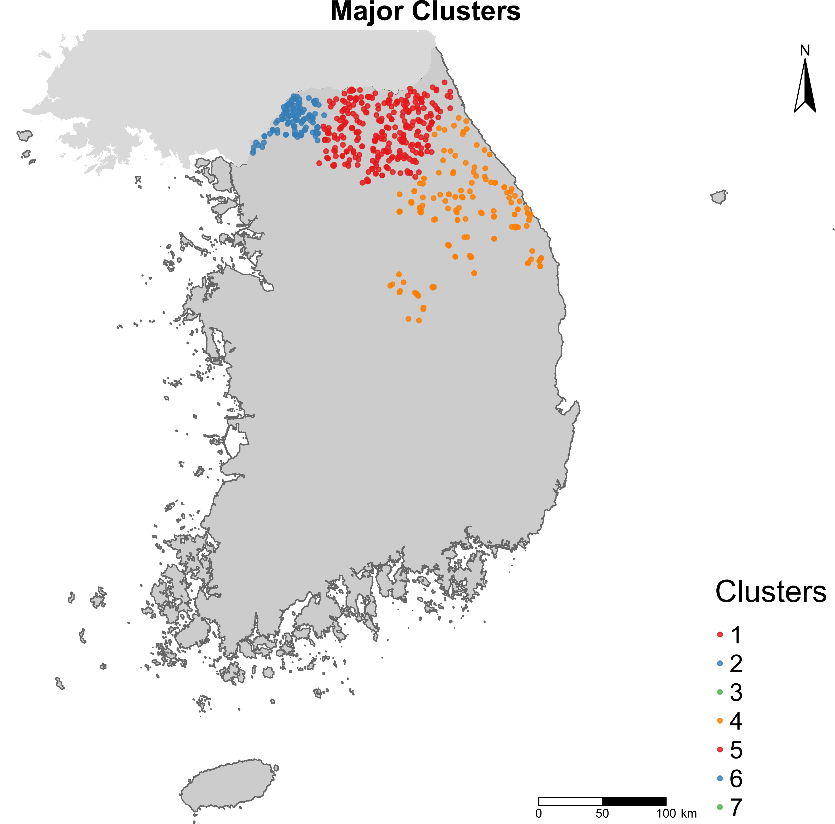


**Figure A1. The identified three major clusters for further analyses under the scenario of the 40 km geographical criterion to select index cases.** Among the seven identified wavefront clusters, cluster 1, 2, and 4, with over 50 cases of wavefront cases, were selected for further analyses.


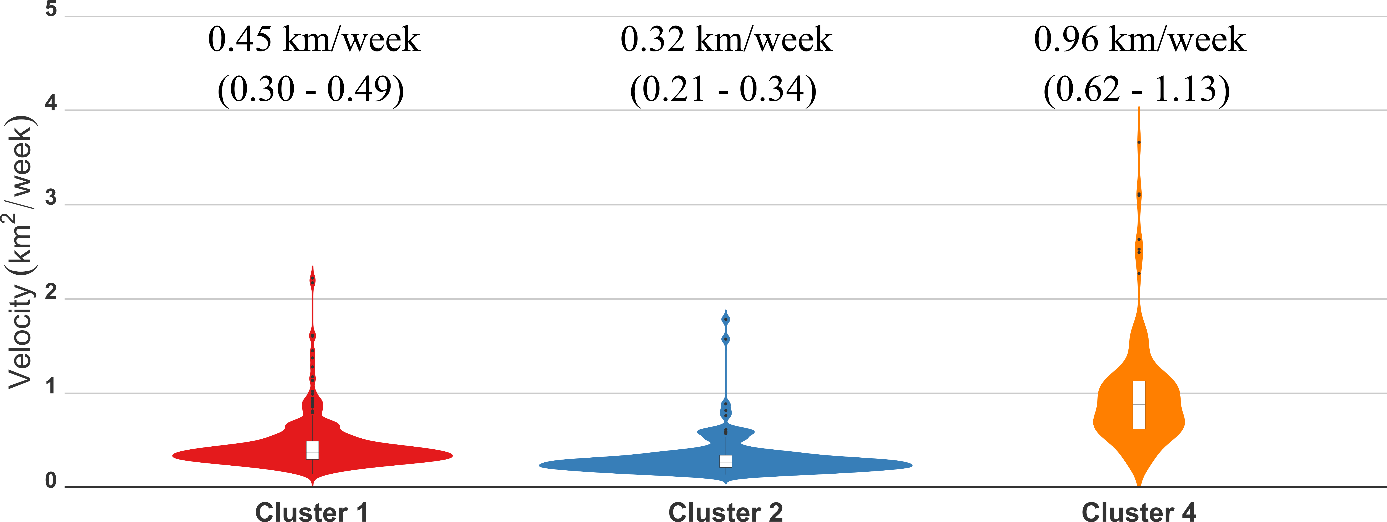
**s**

**Figure A2. Distribution of estimated wavefront velocities for each cluster under the scenario of the 40 km geographical criterion to select index cases.**


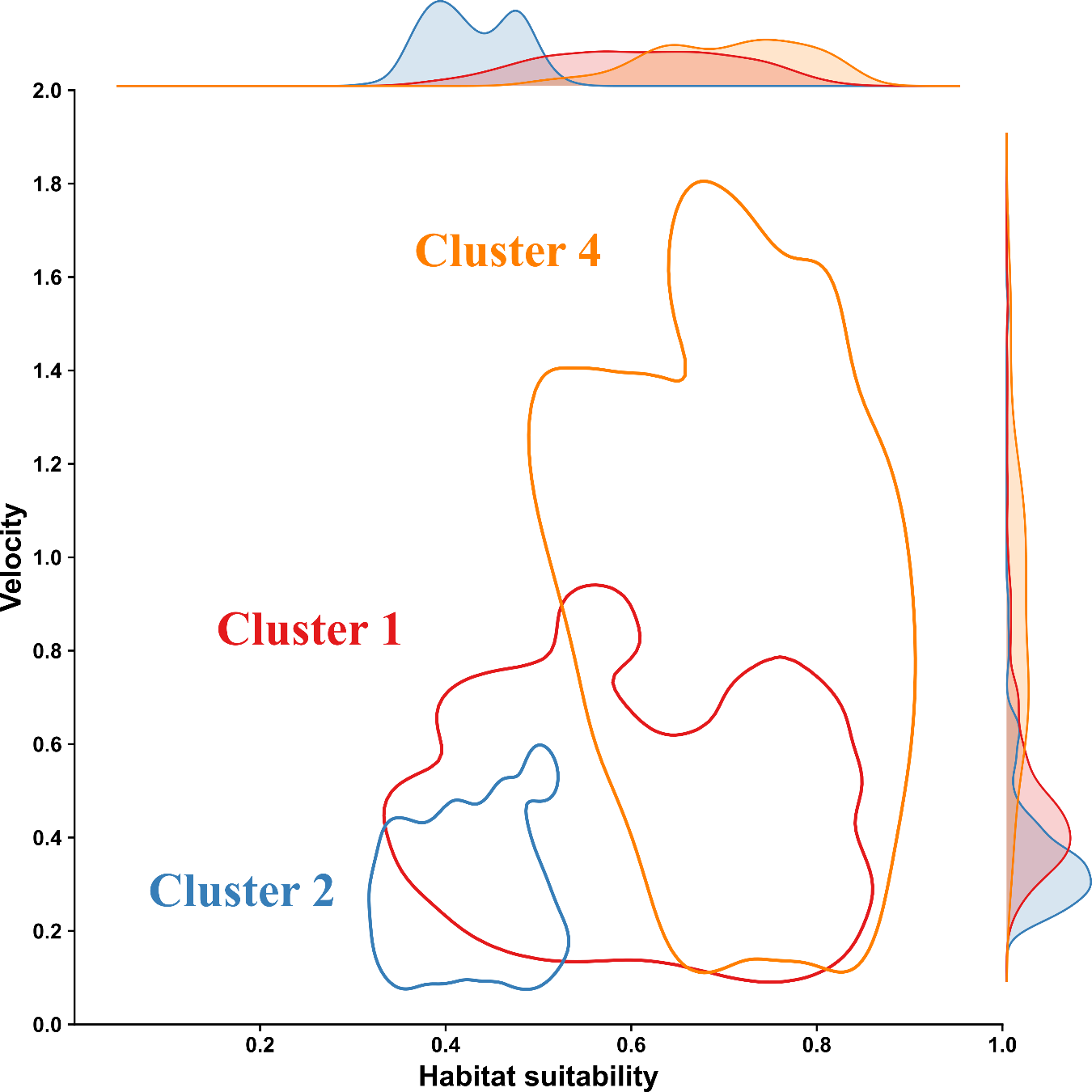


**Figure A3. The joint distribution of the estimated velocity with the function of wild boar habitat suitability in each cluster under the scenario of the 40 km geographical criterion to select index cases.** Each polygon represents the joint distribution of velocity and habitat suitability. Marginal distributions of each variable are plotted in the margins of the plot.


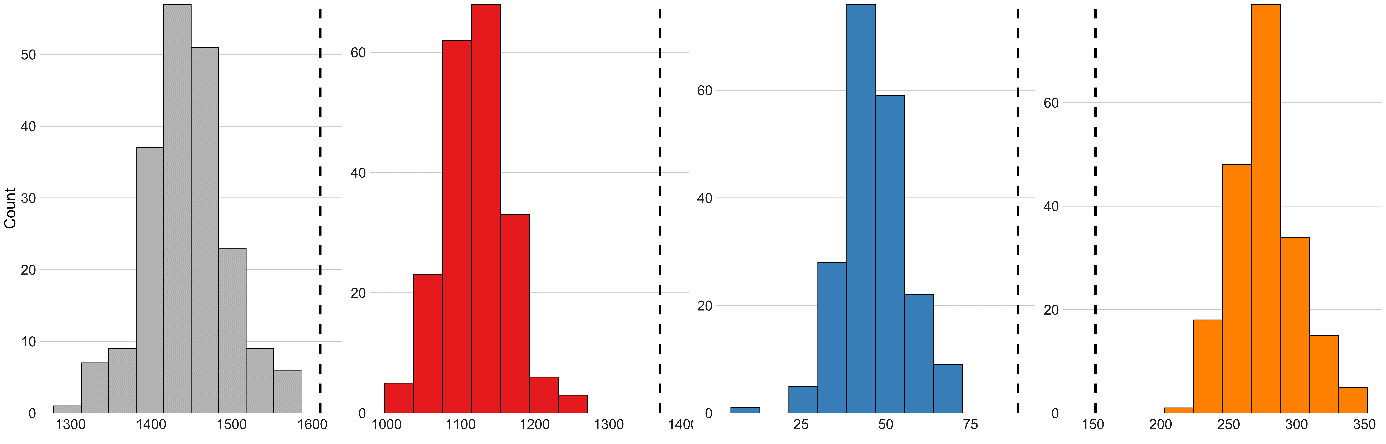


**Figure A4. Sensitivity analysis of national-level and cluster-level *N* statistics under the scenario of the 40 km geographical criterion to select index cases.** Each histogram represents the null distribution of national-level and cluster-level *N* statistics for cluster 1, 2, and 4, in that order. The dashed line indicates the observed *N* statistics. Cluster 2 showed a significant result (*N* = 89, *p*-value< 0.01).

**Table A2. National and Cluster-level *Q* Statistics and Regression Coefficients for Different *k* Values under the Scenario of the 40 km geographical criterion to select index cases.**

| **Cluster** | **k value** | **Regression coefficient** | **Q statistics** |
| --- | --- | --- | --- |
| **National level** | 1 | 406.075 | 0.045 |
|  | 5 | 266.608 | 0.047 |
|  | 10 | 156.767 | 0.005 |
|  | 100 | 11.935 | -0.101 |
|  | 1000 | 0.923 | -0.124 |
|  | 10000 | 0.086 | -0.128 |
| **Cluster 1** | 1 | 696.806 | -0.05 |
|  | 5 | 306.218 | -0.245 |
|  | 10 | 144.107 | -0.346 |
|  | 100 | 5.161 | -0.457 |
|  | 1000 | 0.203 | -0.466 |
|  | 10000 | 0.014 | -0.467 |
| **Cluster 2** | 1 | 62.567 | 0.003 |
|  | 5 | 146.562 | 0.058 |
|  | 10 | 114.49 | 0.086 |
|  | 100 | 14.767 | 0.093 |
|  | 1000 | 1.449 | 0.086 |
|  | 10000 | 0.144 | 0.085 |
| **Cluster 4** | 1 | 51.695 | -0.001 |
|  | 5 | 44.245 | -0.004 |
|  | 10 | 34.656 | -0.008 |
|  | 100 | 0.585 | -0.019 |
|  | 1000 | -0.196 | -0.018 |
|  | 10000 | -0.024 | -0.018 |


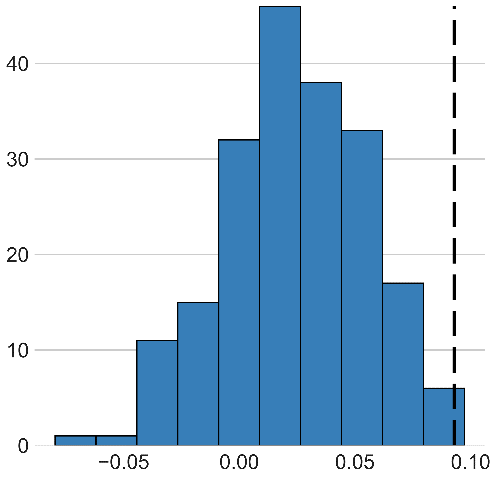


**Figure A5. Sensitivity analysis of national-level and cluster-level *Q* statistics under the scenario of the 40 km geographical criterion to select index cases.** Each histogram represents the null distribution of cluster-level *Q* statistics for cluster 3, with *k* value of 100. Dashed line indicates the observed Q statistics. Each of observed *Q* statistic was 0.047 and 0.093, respectively. *Q* statistic in cluster 2 was significant (*p*-value= 0.01)


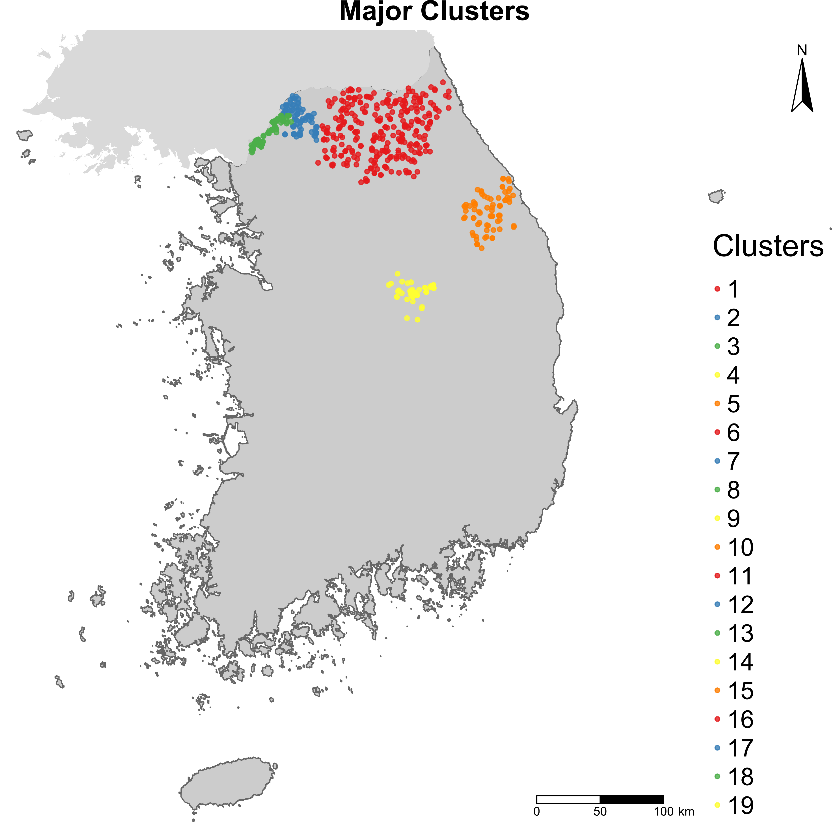


**Figure A6. The identified three major clusters for further analyses under the scenario of the 20 km geographical criterion to select index cases.** Among the 19 identified wavefront clusters, cluster 1, 2, 3, 10, and 14, with over 50 cases of wavefront cases, were selected for further analyses.


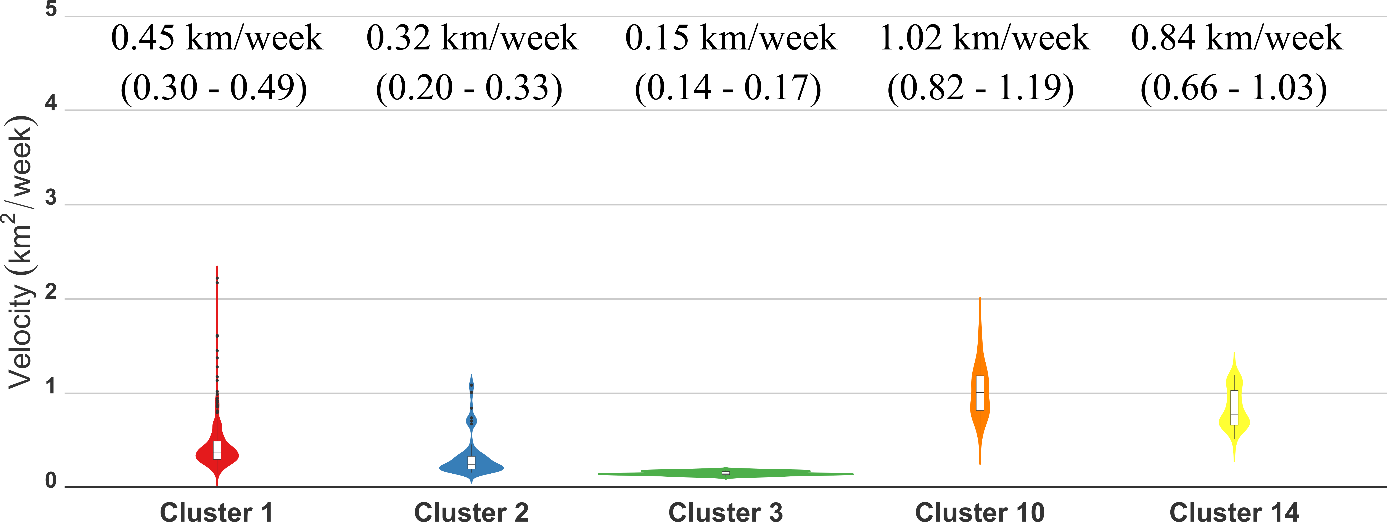


**Figure A7. Distribution of estimated wavefront velocities for each cluster under the scenario of the 20 km geographical criterion to select index cases**


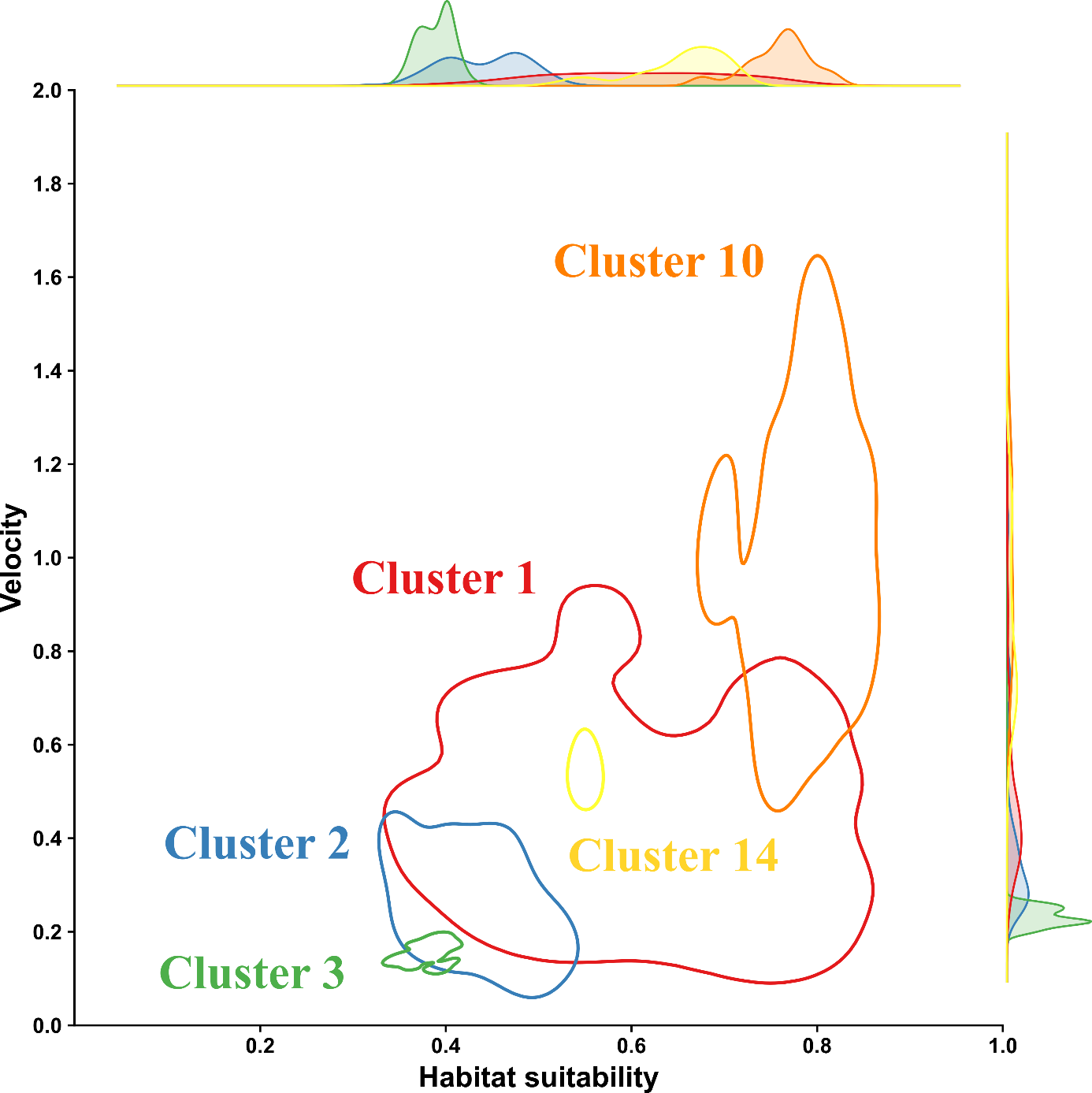


**Figure A8. The joint distribution of the estimated velocity with the function of wild boar habitat suitability in each cluster under the scenario of the 20 km geographical criterion to select index cases.** Each polygon represents the joint distribution of velocity and habitat suitability. Marginal distributions of each variable are plotted in the margins of the plot.


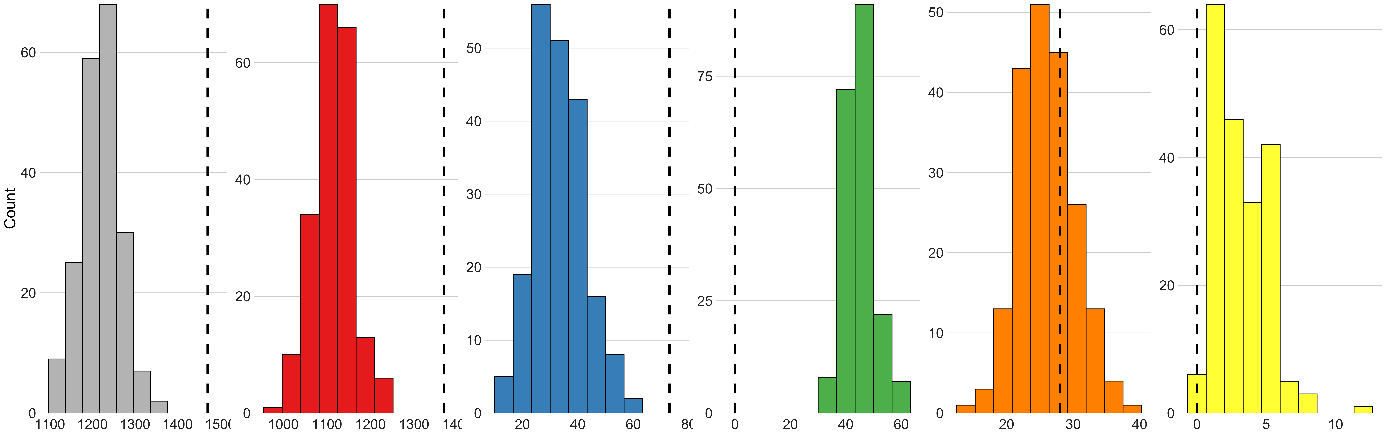


**Figure A9. Sensitivity analysis of national-level and cluster-level *N* statistics under the scenario of the 20 km geographical criterion to select index cases.** Each histogram represents the null distribution of national-level and cluster-level *N* statistics for cluster 1, 2, 10, and 14, in that order. The dashed line indicates the observed *N* statistics. Cluster 3 and 14 showed significant results (*N* = 0, *p*-value< 0.01 and *N* = 0, *p*-value= 0.03, respectively)

**Table A3. National and Cluster-level *Q* Statistics and Regression Coefficients for Different *k* Values under the Scenario of the 20 km of geographical criterion to select index cases.**

| **Cluster** | **k value** | **Regression coefficient** | **Q statistics** |
| --- | --- | --- | --- |
| **National level** | 1 | 810.26 | -0.001 |
|  | 5 | 453.485 | -0.127 |
|  | 10 | 269.316 | -0.242 |
|  | 100 | 22.447 | -0.499 |
|  | 1000 | 1.816 | -0.552 |
|  | 10000 | 0.172 | -0.561 |
| **Cluster 1** | 1 | 695.402 | -0.049 |
|  | 5 | 307.409 | -0.244 |
|  | 10 | 144.873 | -0.347 |
|  | 100 | 5.183 | -0.46 |
|  | 1000 | 0.205 | -0.468 |
|  | 10000 | 0.014 | -0.469 |
| **Cluster 2** | 1 | 667.362 | -0.005 |
|  | 5 | 300.702 | -0.145 |
|  | 10 | 160.783 | -0.235 |
|  | 100 | 13.263 | -0.382 |
|  | 1000 | 1.206 | -0.406 |
|  | 10000 | 0.119 | -0.409 |
| **Cluster 3** | 1 | 509.967 | 0.034 |
|  | 5 | 440.725 | 0.042 |
|  | 10 | 301.491 | -0.025 |
|  | 100 | 8.773 | -0.227 |
|  | 1000 | 0.031 | -0.235 |
|  | 10000 | -0.007 | -0.235 |
| **Cluster 10** | 1 | 231.796 | -0.022 |
|  | 5 | 82.699 | -0.103 |
|  | 10 | 35.652 | -0.132 |
|  | 100 | 2.002 | -0.157 |
|  | 1000 | 0.177 | -0.159 |
|  | 10000 | 0.017 | -0.159 |
| **Cluster 14** | 1 | 344.811 | 0.009 |
|  | 5 | 341.269 | 0.029 |
|  | 10 | 337.459 | 0.044 |
|  | 100 | 233.785 | 0.026 |
|  | 1000 | 28.272 | -0.302 |
|  | 10000 | 2.474 | -0.369 |


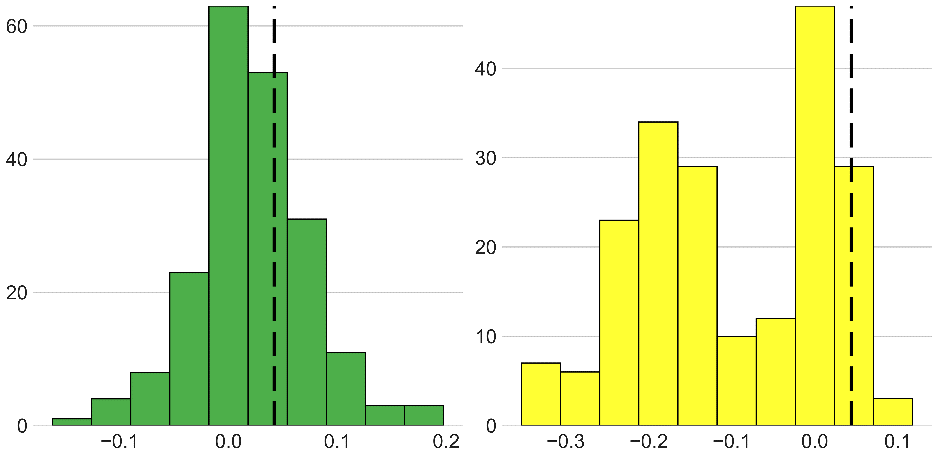


**Figure A10. Sensitivity analysis of cluster-level *Q* statistics under the scenario of the 20 km geographical criterion to select index cases.** Each histogram represents the null distribution of cluster-level *Q* statistics for clusters 3 and 14, with *k* values of 5 and 10, respectively. Dashed line indicates the observed *Q* statistics. Each of observed *Q* statistics was 0.042 and 0.026, respectively, all of which were not significant (*p*-value= 0.3, and *p*-value= 0.07, respectively)

**B. Sensitivity analyses under varying reporting delay scenarios**

The scenarios tested in the sensitivity analyses

1. 50% Shorter Reporting Delay
2. 20% Shorter Reporting Delay
3. 20% Longer Reporting Delay
4. 50% Longer Reporting Delay

The outputs from the sensitivity analysis

1. Wavefront velocity
2. *N* statistic
3. *Q* statistic

**Table B1. Summary of the sensitivity analysis under varying reporting delay scenarios**

|  | Velocity  Mean (IQR) | *N* statistic | | *Q* statistic | |
| --- | --- | --- | --- | --- | --- |
|  |  | National level | Cluster level | National level | Cluster level |
| Baseline | 0.52  (0.25 – 0.62) | Not significant | 3, 5 | Not significant | Not significant |
| -50% | 0.54  (0.24 – 0.58) | Not significant | 3, 5 | Not significant | Not significant |
| -20% | 0.53  (0.25 – 0.62) | Not significant | 3, 5 | Not significant | Not significant |
| +20% | 0.51  (0.26 – 0.63) | Not significant | 3, 5 | Not significant | Not significant |
| +50% | 0.49  (0.26 – 0.59) | Not significant | 3, 5 | Not significant | Not significant |

**
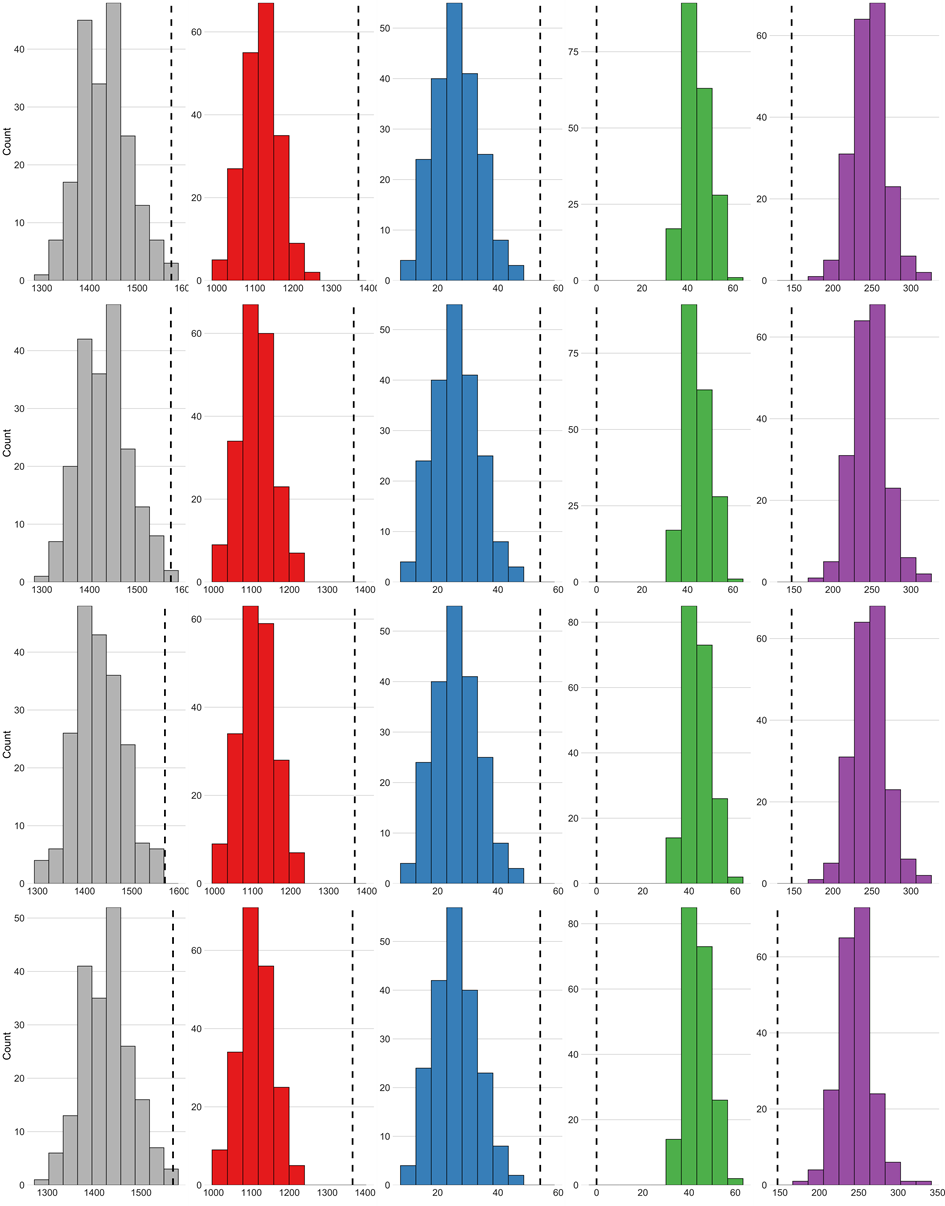
**

**Figure B1. Sensitivity analysis of national-level and cluster-level *N* statistics under the scenario of the varying reporting delay.** From top to bottom, each row presents the results from sensitivity analysis for different reporting delays: a 50% shorter reporting delay, a 20% shorter reporting delay, a 20% longer reporting delay, and a 50% longer reporting delay, respectively. Each column indicates result of national-level *N* statistics, cluster-level *N* statistics in cluster 1, 2, 3, and 5.

**Table B2. National and Cluster-level *Q* Statistics and Regression Coefficients for Different *k* Values under the Scenario of a 50% Shorter Reporting Delay.**

| ***k* value** | **Cluster** | **Regression coefficient** | ***Q* statistics** |
| --- | --- | --- | --- |
| **National level** | 1 | 969.836 | 0.011 |
|  | 5 | 451.501 | -0.192 |
|  | 10 | 223.571 | -0.325 |
|  | 100 | 12.371 | -0.500 |
|  | 1000 | 0.873 | -0.522 |
|  | 10000 | 0.081 | -0.525 |
| **Cluster 1** | 1 | 989.906 | -0.085 |
|  | 5 | 328.795 | -0.379 |
|  | 10 | 127.897 | -0.484 |
|  | 100 | 2.003 | -0.559 |
|  | 1000 | -0.064 | -0.560 |
|  | 10000 | -0.010 | -0.560 |
| **Cluster 2** | 1 | 897.113 | -0.002 |
|  | 5 | 358.785 | -0.147 |
|  | 10 | 181.151 | -0.229 |
|  | 100 | 13.317 | -0.351 |
|  | 1000 | 1.175 | -0.340 |
|  | 10000 | 0.115 | -0.372 |
| **Cluster 3** | 1 | 810.486 | 0.017 |
|  | 5 | 512.675 | -0.071 |
|  | 10 | 248.710 | -0.167 |
|  | 100 | 3.914 | -0.265 |
|  | 1000 | -0.029 | -0.267 |
|  | 10000 | -0.008 | -0.267 |
| **Cluster 5** | 1 | 540.206 | -0.012 |
|  | 5 | 378.318 | -0.120 |
|  | 10 | 213.363 | -0.233 |
|  | 100 | 2.998 | -0.378 |
|  | 1000 | -0.332 | -0.377 |
|  | 10000 | -0.049 | -0.375 |

**Table B3. National and Cluster-level *Q* Statistics and Regression Coefficients for Different *k* Values under the Scenario of a 20% Shorter Reporting Delay.**

| ***k* value** | **Cluster** | **Regression coefficient** | ***Q* statistics** |
| --- | --- | --- | --- |
| **National level** | 1 | 969.863 | 0.012 |
|  | 5 | 451.501 | -0.192 |
|  | 10 | 223.571 | -0.325 |
|  | 100 | 12.371 | -0.500 |
|  | 1000 | 0.873 | -0.522 |
|  | 10000 | 0.081 | -0.525 |
| **Cluster 1** | 1 | 998.970 | -0.083 |
|  | 5 | 330.068 | -0.378 |
|  | 10 | 128.588 | -0.483 |
|  | 100 | 2.055 | -0.559 |
|  | 1000 | -0.059 | -0.560 |
|  | 10000 | -0.010 | -0.560 |
| **Cluster 2** | 1 | 891.166 | -0.002 |
|  | 5 | 358.771 | -0.147 |
|  | 10 | 181.112 | -0.229 |
|  | 100 | 13.308 | -0.351 |
|  | 1000 | 1.174 | -0.340 |
|  | 10000 | 0.115 | -0.372 |
| **Cluster 3** | 1 | 810.485 | 0.017 |
|  | 5 | 512.673 | -0.071 |
|  | 10 | 248.709 | -0.167 |
|  | 100 | 3.914 | -0.265 |
|  | 1000 | -0.029 | -0.267 |
|  | 10000 | -0.008 | -0.267 |
| **Cluster 5** | 1 | 540.206 | -0.012 |
|  | 5 | 378.318 | -0.120 |
|  | 10 | 213.363 | -0.233 |
|  | 100 | 2.998 | -0.378 |
|  | 1000 | -0.332 | -0.377 |
|  | 10000 | -0.049 | -0.375 |

**Table B4. National and Cluster-level *Q* Statistics and Regression Coefficients for Different *k* Values under the Scenario of a 20% Longer Reporting Delay.**

| ***k* value** | **Cluster** | **Regression coefficient** | ***Q* statistics** |
| --- | --- | --- | --- |
| **National level** | 1 | 971.452 | 0.013 |
|  | 5 | 457.054 | -0.185 |
|  | 10 | 228.021 | -0.318 |
|  | 100 | 12.912 | -0.496 |
|  | 1000 | 0.923 | -0.519 |
|  | 10000 | 0.085 | -0.523 |
| **Cluster 1** | 1 | 992.024 | -0.079 |
|  | 5 | 338.844 | -0.368 |
|  | 10 | 135.182 | -0.475 |
|  | 100 | 2.821 | -0.557 |
|  | 1000 | 0.013 | -0.560 |
|  | 10000 | -0.002 | -0.560 |
| **Cluster 2** | 1 | 897.166 | -0.002 |
|  | 5 | 358.771 | -0.147 |
|  | 10 | 181.111 | -0.228 |
|  | 100 | 13.307 | -0.351 |
|  | 1000 | 1.174 | -0.369 |
|  | 10000 | 0.115 | -0.371 |
| **Cluster 3** | 1 | 810.716 | 0.017 |
|  | 5 | 514.151 | -0.068 |
|  | 10 | 250.565 | -0.164 |
|  | 100 | 3.985 | -0.265 |
|  | 1000 | -0.027 | -0.266 |
|  | 10000 | -0.007 | -0.266 |
| **Cluster 5** | 1 | 540.203 | -0.012 |
|  | 5 | 378.312 | -0.119 |
|  | 10 | 213.359 | -0.232 |
|  | 100 | 2.998 | -0.377 |
|  | 1000 | -0.332 | -0.377 |
|  | 10000 | -0.049 | -0.375 |

**Table B5. National and Cluster-level *Q* Statistics and Regression Coefficients for Different *k* Values under the Scenario of a 50% Longer Reporting Delay.**

| ***k* value** | **Cluster** | **Regression coefficient** | ***Q* statistics** |
| --- | --- | --- | --- |
| **National level** | 1 | 969.833 | 0.012 |
|  | 5 | 451.501 | -0.192 |
|  | 10 | 223.571 | -0.325 |
|  | 100 | 12.371 | -0.500 |
|  | 1000 | 0.873 | -0.522 |
|  | 10000 | 0.081 | -0.525 |
| **Cluster 1** | 1 | 988.970 | -0.083 |
|  | 5 | 330.068 | -0.378 |
|  | 10 | 128.588 | -0.483 |
|  | 100 | 2.055 | -0.559 |
|  | 1000 | -0.059 | -0.560 |
|  | 10000 | -0.001 | -0.560 |
| **Cluster 2** | 1 | 897.166 | -0.002 |
|  | 5 | 358.771 | -0.147 |
|  | 10 | 181.111 | -0.229 |
|  | 100 | 13.308 | -0.351 |
|  | 1000 | 1.174 | -0.370 |
|  | 10000 | 0.115 | -0.372 |
| **Cluster 3** | 1 | 810.485 | 0.017 |
|  | 5 | 512.673 | -0.071 |
|  | 10 | 248.709 | -0.167 |
|  | 100 | 3.914 | -0.265 |
|  | 1000 | -0.029 | -0.267 |
|  | 10000 | -0.008 | -0.267 |
| **Cluster 5** | 1 | 540.206 | -0.012 |
|  | 5 | 378.318 | -0.120 |
|  | 10 | 213.363 | -0.233 |
|  | 100 | 2.998 | -0.378 |
|  | 1000 | -0.332 | -0.377 |
|  | 10000 | -0.049 | -0.375 |


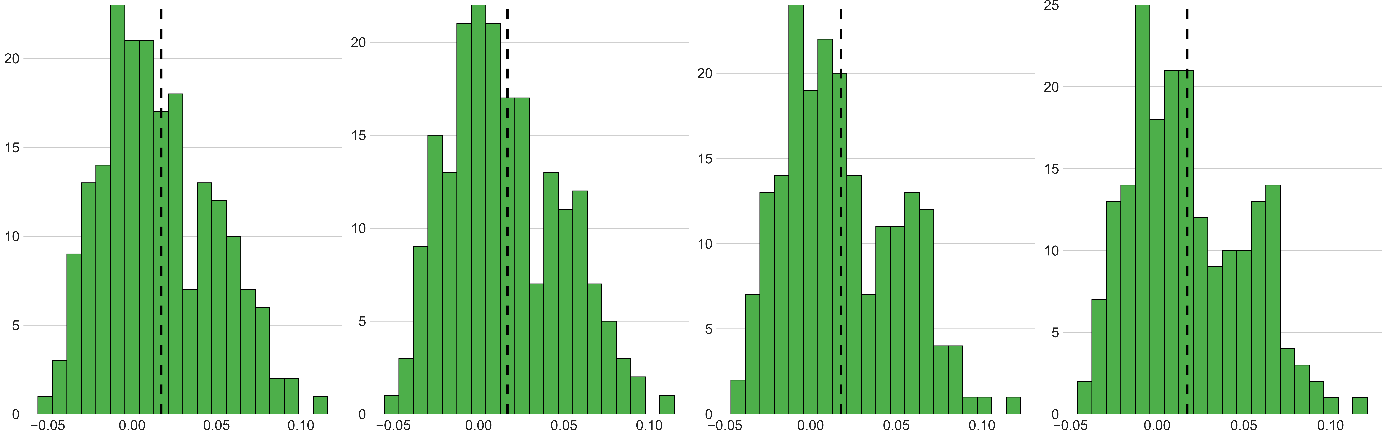


**Figure B2. Sensitivity analysis of *Q* statistics for cluster 3 under the scenario of varying reporting delay.** Each histogram presents the cluster level *Q* statistics with *k* value of 1 under the scenarios of a 50% shorter reporting delay, a 20% shorter reporting delay, a 20% longer reporting delay, and a 50% longer reporting delay, respectively. Each horizontal dashed line indicates the observed *Q* statistic under each scenario.

**C. Sensitivity analyses under varying installation times of fences**

The scenarios tested in the sensitivity analyses

1. Starting Date of fence installation
2. Ending Date of fence installation

The outputs from the sensitivity analysis

1. *N* statistic
2. *Q* statistic

**Table C1. Summary of the sensitivity analysis under varying reporting delay scenarios**

|  | *N* statistic | | *Q* statistic | |
| --- | --- | --- | --- | --- |
|  | National level | Cluster level | National level | Cluster level |
| Baseline | Not significant | 3, 5 | Not significant | Not significant |
| Starting date | Not significant | 3, 5 | Not significant | Not significant |
| Ending date | Not significant | 3, 5 | Not significant | Not significant |


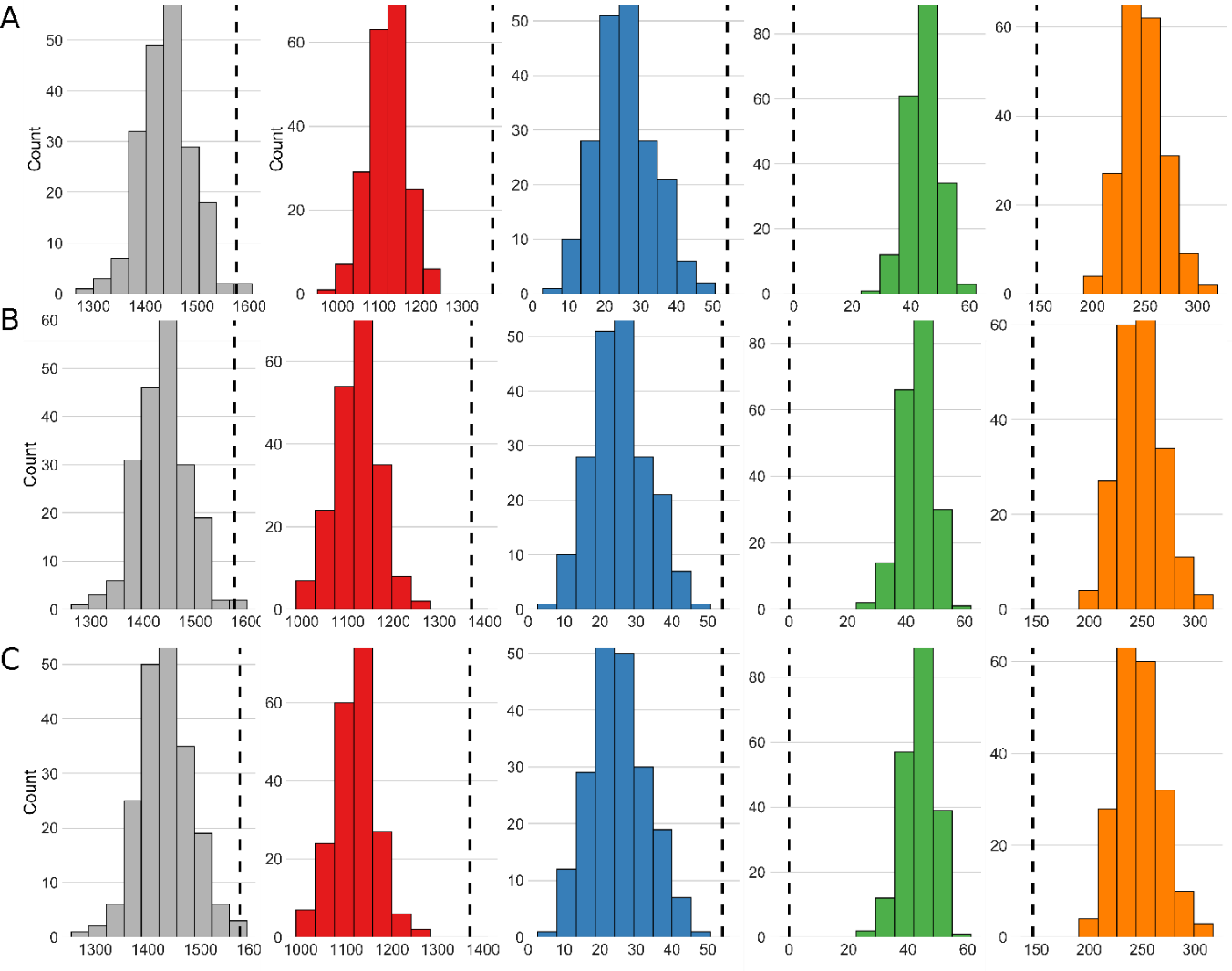


**Figure C1. Sensitivity analysis of national-level and cluster-level *N* statistics under the scenario of the different fencing installation time.** Each row indicates the results of sensitivity analysis based on different fencing installation time. A) Results based on the starting date of installation, B) median date between starting and finishing date of installation and C) ending date of installation. Each column indicates result of national-level *N* statistics, cluster-level *N* statistics in cluster 1, 2, 3, and 5.

**Table C2. National and Cluster-level *Q* Statistics and Regression Coefficients for Different *k* Values under the Scenario of the starting date of fence installation.**

| **Cluster** | **k value** | **Regression coefficient** | **Q statistics** |
| --- | --- | --- | --- |
| **National level** | 1 | 986.72 | -0.023 |
|  | 5 | 428.46 | -0.274 |
|  | 10 | 202.424 | -0.409 |
|  | 100 | 9.374 | -0.564 |
|  | 1000 | 0.577 | -0.58 |
|  | 10000 | 0.051 | -0.582 |
| **Cluster 1** | 1 | 980.317 | -0.092 |
|  | 5 | 310.9 | -0.394 |
|  | 10 | 114.923 | -0.494 |
|  | 100 | 0.576 | -0.556 |
|  | 1000 | -0.198 | -0.555 |
|  | 10000 | -0.024 | -0.555 |
| **Cluster 2** | 1 | 889.197 | -0.001 |
|  | 5 | 357.011 | -0.143 |
|  | 10 | 181.104 | -0.225 |
|  | 100 | 13.524 | -0.349 |
|  | 1000 | 1.198 | -0.368 |
|  | 10000 | 0.118 | -0.37 |
| **Cluster 3** | 1 | 803.18 | 0.016 |
|  | 5 | 506.241 | -0.073 |
|  | 10 | 244.644 | -0.168 |
|  | 100 | 3.592 | -0.265 |
|  | 1000 | -0.057 | -0.267 |
|  | 10000 | -0.01 | -0.266 |
| **Cluster 5** | 1 | 525.286 | -0.012 |
|  | 5 | 366.864 | -0.116 |
|  | 10 | 206.329 | -0.225 |
|  | 100 | 2.679 | -0.362 |
|  | 1000 | -0.345 | -0.361 |
|  | 10000 | -0.05 | -0.359 |

**Table C2. National and Cluster-level *Q* Statistics and Regression Coefficients for Different *k* Values under the Scenario of the ending date of fence installation.**

| **Cluster** | **k value** | **Regression coefficient** | **Q statistics** |
| --- | --- | --- | --- |
| **National level** | 1 | 992.606 | -0.017 |
|  | 5 | 443.984 | -0.257 |
|  | 10 | 214.554 | -0.393 |
|  | 100 | 10.779 | -0.557 |
|  | 1000 | 0.706 | -0.576 |
|  | 10000 | 0.064 | -0.578 |
| **Cluster 1** | 1 | 990.404 | -0.082 |
|  | 5 | 334.238 | -0.372 |
|  | 10 | 131.686 | -0.476 |
|  | 100 | 2.405 | -0.555 |
|  | 1000 | -0.027 | -0.557 |
|  | 10000 | -0.007 | -0.556 |
| **Cluster 2** | 1 | 893.156 | 0.001 |
|  | 5 | 358.312 | -0.144 |
|  | 10 | 180.995 | -0.227 |
|  | 100 | 13.284 | -0.352 |
|  | 1000 | 1.171 | -0.371 |
|  | 10000 | 0.115 | -0.373 |
| **Cluster 3** | 1 | 802.716 | 0.017 |
|  | 5 | 506.807 | -0.07 |
|  | 10 | 246.343 | -0.166 |
|  | 100 | 3.69 | -0.265 |
|  | 1000 | -0.056 | -0.267 |
|  | 10000 | -0.01 | -0.266 |
| **Cluster 5** | 1 | 526.686 | -0.011 |
|  | 5 | 369.333 | -0.113 |
|  | 10 | 208.026 | -0.223 |
|  | 100 | 2.728 | -0.362 |
|  | 1000 | -0.344 | -0.362 |
|  | 10000 | -0.05 | -0.359 |


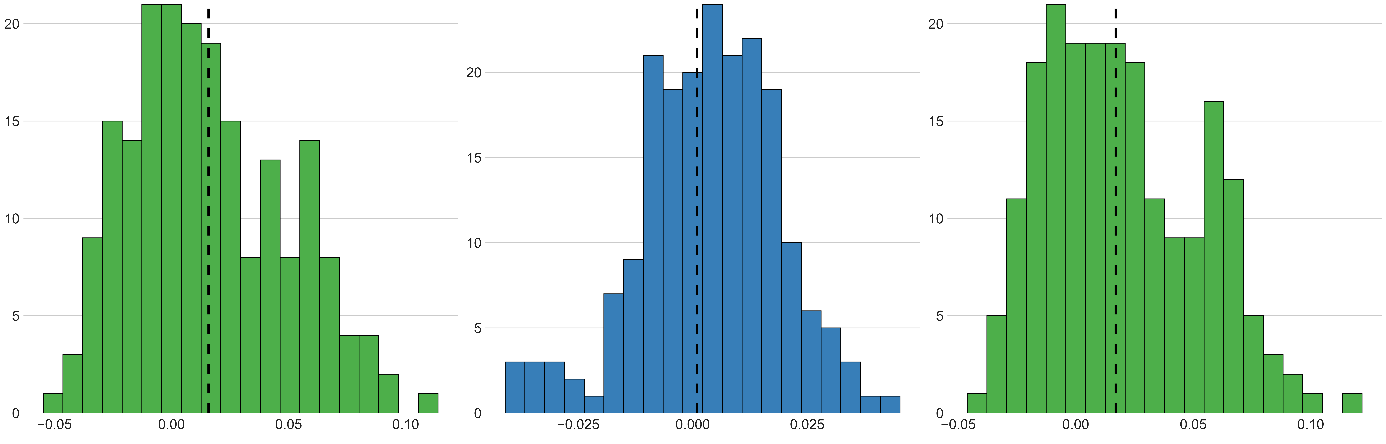


**Figure C2. Sensitivity analysis of cluster-level *Q* statistics under the scenario of the different fencing installation time.** Each histogram represents the null distribution of cluster-level *N* statistics for cluster 3 with *k* value of 1 under the scenario of starting date of fence installation, and cluster 2 and 3 with *k* value of 1 under the scenarios of ending date of fence installation, in that order. The dashed line indicates the observed *N* statistics.
