# Supplementary material for: A spatially-heterogeneous impact of fencing on the African swine fever wavefront in the Korean wild boar population
Source: Vet Res. 2024 Dec 18;55:163. doi: 10.1186/s13567-024-01422-7 (PMC11654197; doi:10.1186/s13567-024-01422-7)

**Additional file 8 The distribution of selected wavefront cases in each cluster and installed fencing.**


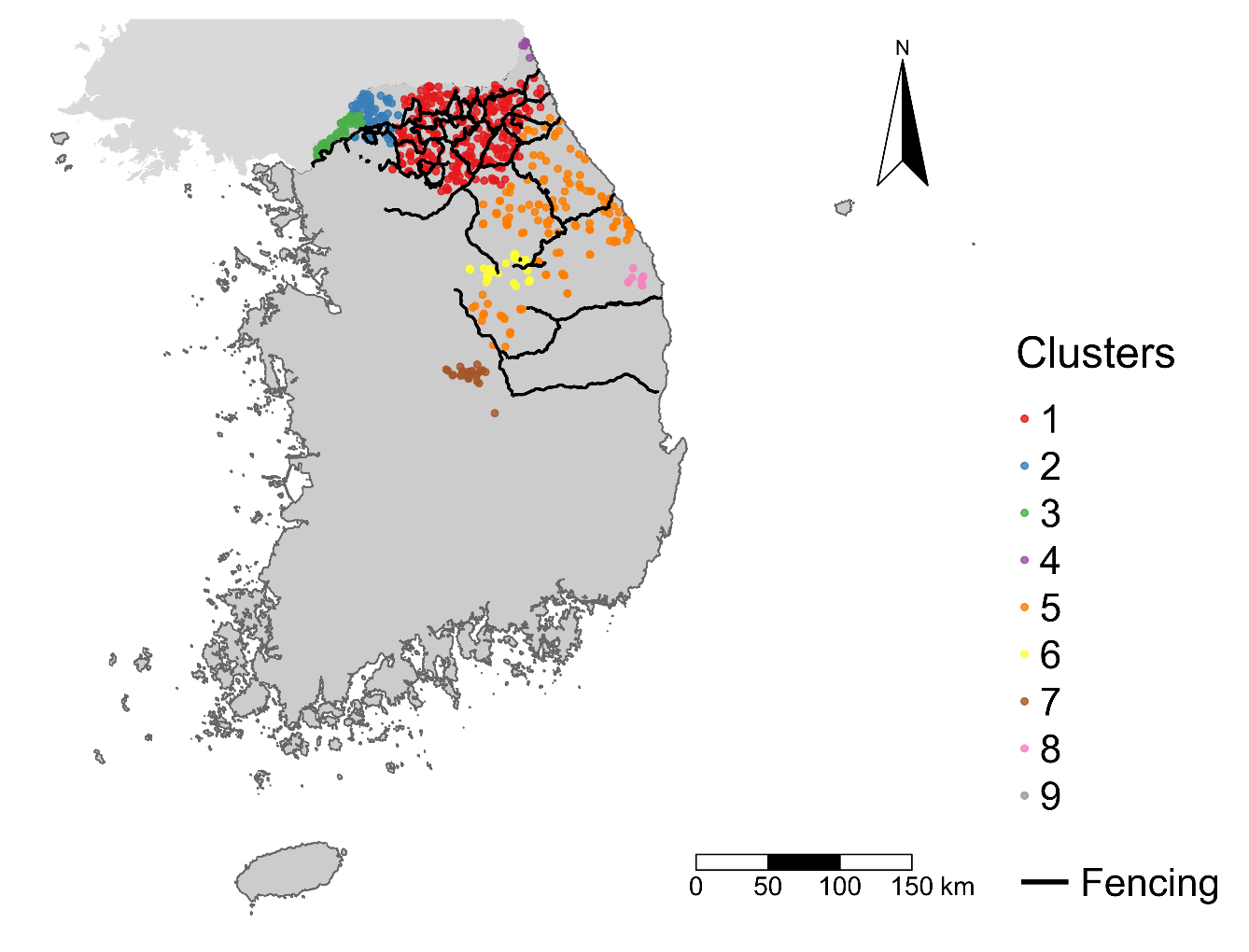

Supplement: Supplementary file 8 — Additional file 8: The distribution of selected wavefront cases in each cluster and installed fencing. [file 13567_2024_1422_MOESM8_ESM.docx]
